# Next Generation Sequencing and Transcriptome Analysis Predicts Biosynthetic Pathway of Sennosides from Senna (*Cassia angustifolia* Vahl.), a Non-Model Plant with Potent Laxative Properties

**DOI:** 10.1371/journal.pone.0129422

**Published:** 2015-06-22

**Authors:** Nagaraja Reddy Rama Reddy, Rucha Harishbhai Mehta, Palak Harendrabhai Soni, Jayanti Makasana, Narendra Athamaram Gajbhiye, Manivel Ponnuchamy, Jitendra Kumar

**Affiliations:** ICAR-Directorate of Medicinal and Aromatic Plants Research (DMAPR), Anand, Gujarat, India; Louisiana State University Agricultural Center, UNITED STATES

## Abstract

Senna (*Cassia angustifolia* Vahl.) is a world’s natural laxative medicinal plant. Laxative properties are due to sennosides (anthraquinone glycosides) natural products. However, little genetic information is available for this species, especially concerning the biosynthetic pathways of sennosides. We present here the transcriptome sequencing of young and mature leaf tissue of *Cassia angustifolia* using Illumina MiSeq platform that resulted in a total of 6.34 Gb of raw nucleotide sequence. The sequence assembly resulted in 42230 and 37174 transcripts with an average length of 1119 bp and 1467 bp for young and mature leaf, respectively. The transcripts were annotated using NCBI BLAST with ‘green plant database (txid 33090)’, Swiss Prot, Kyoto Encylcopedia of Genes & Genomes (KEGG), Cluster of Orthologous Gene (COG) and Gene Ontology (GO). Out of the total transcripts, 40138 (95.0%) and 36349 (97.7%) from young and mature leaf, respectively, were annotated by BLASTX against green plant database of NCBI. We used InterProscan to see protein similarity at domain level, a total of 34031 (young leaf) and 32077 (mature leaf) transcripts were annotated against the Pfam domains. All transcripts from young and mature leaf were assigned to 191 KEGG pathways. There were 166 and 159 CDS, respectively, from young and mature leaf involved in metabolism of terpenoids and polyketides. Many CDS encoding enzymes leading to biosynthesis of sennosides were identified. A total of 10,763 CDS differentially expressing in both young and mature leaf libraries of which 2,343 (21.7%) CDS were up-regulated in young compared to mature leaf. Several differentially expressed genes found functionally associated with sennoside biosynthesis. CDS encoding for many CYPs and TF families were identified having probable roles in metabolism of primary as well as secondary metabolites. We developed SSR markers for molecular breeding of senna. We have identified a set of putative genes involved in various secondary metabolite pathways, especially those related to the synthesis of sennosides which will serve as an important platform for public information about gene expression, genomics, and functional genomics in senna.

## Introduction

Senna (*Cassia angustifolia* Vahl. is synonymous with *Senna alexandria* Mill.) [1], popular as “Tirunelveli senna” is used worldwide as natural laxative. The drug senna is mentioned in various texts of indigenous systems of medicine (Ayurveda, Siddha Unani and Homoeopathy) in India, pharmacopeias of United States, United Kingdom, Germany, and other counties [2–5]. Tirunelveli is a place in south India where senna was introduced in India for the first time in the mid-eighteenth century and it is extensively cultivated, processed, and exported to various countries under the brand name “Tirunelveli senna” hence the name. The drug senna is widely used as a purgative, laxative, expectorant, wound dresser, antidysentric, and carminative. Senna leaves are commonly used as natural laxative both in modern as well as in traditional systems of medicine. The calcium sennoside is a popular form of dispensation useful in habitual constipation in modern medicine [6]. However, leaves are having international demand and preferred as ingredient of herbal tea in Europe [7].

Senna plant is a small, 1–2 m height under-shrub and belongs to family Caesalpiniaceae. The stem is erect, smooth, and pale green to light brown with long spreading branches. Leaves are compound with four to eight pairs of leaflets. It is mucilaginous with sweet taste and peculiar odour. The flowers are small and yellow. The pods are broadly oblong, about 5–8 cm long and 2–3 cm broad bearing about six to nine seeds. *Cassia angustifolia* is cultivated mainly in India and Pakistan [6] and is native to tropical Africa and cultivated in Egypt, Sudan, and elsewhere [8].

Sennosides are the anthraquinone glycosides; (four types of Sennoside A, B, C, and D) found in large quantities in leaves (2.0–3.0%) and pods (3.0–4.0%) of Senna (*Cassia angustifolia*) [6]. Sennosides A and B are homo-dianthrones (dimers of two similar anthrone moieties) of Rhein anthrone whereas Sennoside C and D are hetro-dianthrones of Rhein and Aloe-emodin anthrones. Sennoside A and B contribute for around 80% of the biological activity of senna [9,10]. Sennosides act on the large intestine to stimulate peristalsis (the muscular activity of the colon leading to elimination) [11–13]. In plant, the sennosides are absent in fresh parts and form only during post harvest drying [14]. The dried leaflets and pods are main tissue used in herbal medicine and the pharmaceutical industry.

The biosynthetic pathway leading to biosynthesis of sennosides in plants is unknown and relevant pathways are difficult to elucidate. The knowledge of sennoside biosynthesis in the plants is derived from studies on Anthraquinone biosynthesis in other species. Biosynthesis of anthraquinones was studied in the plant of family Rubiaceae such as those for *Morinda*, *Rubia* and *Galium* species [15–17]. One of the remarkable features of Anthraquinone biosynthesis in higher plants is that they are derived from a variety of different precursors and pathways [15,18] and hence difficult to elucidate. Anthraquinone are thought to be biosynthesized in the plant by a combination of isochorismate and plastidic hemiterpenoid 2-C-methyl-D-erthriol-4-phosphate (MEP) pathways [19,20]. The mevalonate pathway is an important cellular metabolic pathway present in all higher eukaryotes and many bacteria. The products of MEP and MVA pathway i e., 3,3-dimethylallyl diphosphate (DMAPP) and isopentenyl diphosphate (IPP) are the important intermediates in production of many secondary metabolites in plants [21,22]. However, the enzymes and genes involved in the biosynthesis of these complex molecules are largely uncharacterized. The backbone of anthraquinones are synthesized via the isochorismate and MVA/MEP pathway. Anthraquinone is made up of three benzene rings namely A, B and C. The rings A and B of anthraquinones are derived from 1,4-dihydroxy-2- naphthoic acid via isochorimmic acid and α-ketoglutaric acid, whereas ring C of anthraquinones is derived from isopentenyl diphosphate (IPP)/3,3-dimethylallyl diphosphate (DMAPP) via the MVA/MEP pathway [15,18]. Only a limited number of genes encoding for enzymes of each step have been identified and characterized that play an important role in the modification of the anthraquinone backbone structure. A few cloned and characterized genes of the MEP pathway in plants were reported [23] like., the gene DXS encoding 1-deoxy-d-xylulose -5-phosphate synthase (DXS, EC 4.1.3.37) enzyme in *Arabidopsis* [24] and in 15 other plants species [23], DXR gene encoding 1-deoxy-d-xylulose 5-phosphate reductase (DXR, EC 1.1.1.267) enzyme in *Arabidopsis* [25] and in 19 other plants species [23], ISPD gene encoding 4-Diphosphocytidyl-2C-methyl-d-erythritol 4-phosphate synthase (CMS, EC 2.7.7.60) enzyme in *Arabidopsis* [26] and other plant species [23], ISPE gene encoding 4-(cytidine-5’-diphospho)-2-C-methyl-d-Erythritol kinase (CMK, EC 2.7.1.148) enzyme in *Arabidopsis* [27], *Lycopersicon* [28], *Mentha* [29] and Rice [30], ISPF gene encoding 2C-methyl-d-erythritol 2,4-cyclodiphosphate synthase (MCS, EC 4.6.1.12) in *Arabidopsis* [31], Ginkgo [32], Rice [33] and *Taxus* [34], gcpE/ispG gene that encodes 4-Hydroxy-3-methylbut-2-enyl-diphosphate synthase (HDS, EC, 1.17.1.2) in *Nicotiana* [35] and Rice [36] and HDR/ISPH gene encoding 1-Hydroxy-2-methyl-butenyl 4-diphosphate reductase (HDR, EC 1.17.1.2) enzyme in *Arabidopsis* [37] and *Nicotiana* [35] were cloned in plants. Traditional approaches to gene cloning often require the isolation and partial sequencing of the appropriate enzyme in an attempt to obtain a genetic probe. This is often expensive, difficult, time-consuming, and futile. Recently, genome-wide studies of model plant species have resulted in an explosive increase in our knowledge and capacity to understand, basic biological processes. In the post genomic era, ‘next-generation sequencing (NGS)’ technology has revolutionized the pace of DNA sequencing in plants and animals. NGS technology, allow holistic profiling of RNA expression [38,39] in non-model plant species in which limited molecular genetics studies have been performed. RNA sequencing (RNA-seq), provides whole-transcriptome expression profiles of selected plant tissues or cells, thereby permitting the integrated analysis of transcriptomes and metabolomes in any plant species. Transcriptome analysis followed by identification of potential candidate genes involved in the secondary metabolic pathway will lead to a better understanding of biosynthesis, regulation and chemical diversity of secondary metabolites in a plant species. Transcriptome analysis by using NGS sequencing has been used extensively to unravel genes encoding enzyme involved in various steps of biosynthetic pathways of active principles in medicinal plants. Some includes the identification of genes encoding metabolic steps involved in the biosynthetic pathway of artemisinin in *Artemisia annua* [40,41], withanolides in *Withania somnifera* [42,43], cannabinoids in *Cannabis sativa* [44], ginsenosides in *Panax ginseng* [45], glycyrrhizin in *Glycyrrhiza uralensis* [46], picrosides in *Picrorhiza kurrooa* [47], hypericin in *Hypericum perforatum* [48], steroidal saponins in *Chlorophytum borivilianum* [49], camptothecin and anthraquinones in *Ophiorrhiza pumila* [50] and steroidal sapogenin biosynthesis in *Asparagus racemosus* [51].

Simple sequence repeats (SSRs), also termed microsatellites, are nucleotide motifs consisting of tandem repeats of two to six base pairs. SSRs are ubiquitous and are found in both protein coding and non-coding regions affecting gene expression [52]. They are favoured for a variety of applications in plant breeding because of their multi-allelic nature, reproducibility, co-dominant inheritance, high abundance, and extensive genome coverage [53]. These markers are used in high-throughput genotyping and thus in the development of high density genetic maps, gene mapping, and marker-assisted selection (MAS). The SSRs from expressed sequence tags (ESTs) (EST-SSRs) are more likely to be tightly associated with the trait and show high cross species transferability [54].

In the present study, in the well known medicinal plant senna, for the first time, we performed a paired-end transcriptome sequencing of young and mature leaf tissues differing for sennoside content using NGS technology. The main objective of our study is to identify candidate genes encoding the enzymes involved in the biosynthetic pathway of sennosides in senna. Our ultimate goal is to engineer the biosynthetic pathways for enhanced production of sennosides. Through our combined analyses, we identified differentially expressed transcripts that are presumed to be associated with the biosynthesis of sennosides. These data sets are useful resources for further studies of the molecular genetics and functional genomics of this species. The enzyme/transcripts identified will also serve the purpose of engineering of anthraquinone biosynthesis in other medicinal plants.

## Material and Methods

### Estimation of sennoside content

#### Chemicals and materials

Sennoside content was estimated using High-performance liquid chromatography (HPLC) method. Reference standards of sennoside-A (purity 96%) and Sennoside-B (purity 94.5%) (Sigma-Aldrich, Bangalore, India), and HPLC grade methanol (Merck Specialties Pvt. Ltd, Mumbai, India) andultra pure distilled water with resistivity greater than 18 MΩ were used. Samples and solutions were filtered with 0.45µ membrane filters, while solvents were degassed prior to use. Leaf samples were collected at flowering from *Cassia angustifolia* (var. sona) plants grown in the experimental farm at the ICAR-Directorate of Medicinal and Aromatic Plants Research (ICAR-DMAPR), Anand, Gujarat in the year 2013. The pure and homozygous seed of ‘sona’ variety was maintained through inbreeding. The ICAR-DMAPR, Anand is located between 22.5^o^ N latitude and 73.0^o^ E longitude, having about 800 mm annual rainfall. Top 25 fresh leaves were collected in triplicates, dried, and powdered separately were used for estimation of sennoside content.

#### Sample preparation

For quantitative estimation of sennosides, the powdered sample of dry leaves (100 mg) was extracted in 20 ml of 70% methanol in water, by sonication for 10 min. The samples were filtered through 0.45 µm membrane before injection into the chromatography system.

#### HPLC parameters

A modular HPLC (Shimadzu Corporation, Kyoto, Japan), Liquid chromatography (LC) system consisting of two LC-20AD pumps, SPD-20A UV-visible detector at 270 nm, DGU-20A3 degasser, SIL-20AC HT autosampler, a CTO-10ASvp column oven, CBM-20 communications bus module were used for chromatographic separation of analytes on a Grace Alltima (100 × 4.6 mm, 3 µm) analytical column (Crawford Scientific, Scotland, UK). The mobile phase consisted of methanol and 1.25% acetic acid in water, in gradient system, at a flow rate of 1.0 mL/min. The column temperature was maintained at 40^°^C for better resolution and the sample injection volume was kept at 10µL.

### Transcriptome sequencing and analysis

#### cDNA Library preparation and sequencing

Total RNA was isolated from the two different leaf samples (fresh young leaves-top 3 and mature leaves-7^th^ leaf from top) using RaFlex Total RNA isolation Kit (Merck Millipore, Massachusetts, USA) by following the standard protocol described by the manufacturer.

The total RNA was dissolved in Nuclease-Free Water (Ambion, USA), and the purity of RNA was verified by measuring Optical Density (OD) Absorption Ratio (A_260/280_) using Nanodrop-8000 spectrophotometer (Thermo Scientific, Wilmington, DE, USA). The quality and integrity of the total RNA was checked using 1% denaturing agarose gel electrophoresis and by visualization under UV light for the presence of intact 28S and 18S bands. The total RNA was quantified using the Qubit Flurometer with Quant-it dsDNA HS kit (Invitrogen). The paired-end cDNA sequencing libraries were prepared using 4 µg of total RNA per sample using TruSeq RNA Sample Preparation V2 Kit (Illumina, San Diego, California, USA) as per manufacturer’s protocol. Library was qualified on Agilent 2100 bioanlyzer using High Sensitivity DNA Chip (Agilent Technologies, CA, United States) for mean size distribution, which was 310 basepair.

#### Preprocessing RNA-Seq data

The next generation sequencing for young and matured leaf total RNA were performed using paired-end (PE) 2x150 bp library on Miseq platform (Illumina, San Diego, California, USA). The raw data was filtered using Trimmomatic v0.30 [55]. Per Base Sequence Quality Score (Q) or Phred quality score Q ≥20 was considered (S1 Fig). High quality data of young and mature leaf plant samples were assembled separately using Trinity RNA-Sequence assembler (Version 2013) [56] on optimized parameters (K mer size for the assembly was set to 25). Further the assembled transcript contigs were validated using CLC Genomics workbench (CLC Bio, Boston, MA 02108 USA) by mapping high quality reads back to the assembled transcript contigs. ORF-Predictor [57], an online tool, was used on default parameters to identify the coding DNA sequences (CDS) from assembled transcript contigs. GC counts of transcripts was determined using a custom-made perl script.

#### Functional annotations

The functional annotation was performed by aligning coding DNA sequence (CDS) to NCBI ‘green plant database (txid 33090)’ database using basic local alignment search tool (BLASTX) [58] with an E-value threshold of 1e^-06^ and GO assignments were used to classify the functions of the predicted CDS. The GO mapping also provided ontology of defined terms representing gene product properties which were grouped into three main domains: biological process (BP), molecular function (MF) and cellular component (CC). GO mapping was carried out in order to retrieve GO terms for all the BLASTX functionally annotated CDS. The GO mapping used defined criteria to retrieve GO terms for annotated CDS which included use of BLASTX result accession IDs to retrieve gene names or symbols, UniProt IDs and direct search in the dbxref table of GO database. Identified gene names or symbols were then searched in the species specific entries of the gene-product tables of GO database. UniProt IDs made use of protein information resource (PIR) which includes protein sequence database (PSD), UniProt, SwissProt, TrEMBL, RefSeq, GenPept, and PDB databases. Gene Ontology analysis helps in specifying all the annotated nodes comprising of GO functional groups. CDS were compared against the COG (Clusters of Orthologous Groups) database for the analysis of phylogenetically widespread domain families. CDS were compared against Pfam database for higher-level groupings of related protein families, known as clans and the identification of domains that occurs within proteins. BLASTX was used against uniprot-swissprot database with cut-off e-value 1e-6 to annotate predicted CDS against protein. To assign the putative transcription factor terms to the contigs, the transcripts were aligned to the Plant Transcription Factor Database (http://planttfdb.cbi.pku.edu.cn/) using BLAST X. Cytochrome P450 (CYPs) were identified in the annotated data set using in-house DATA mining tools.

#### Pathway mapping of CDS by KEGG.

Ortholog assignment and mapping of the CDS to the biological pathways were performed using kyoto encyclopedia of genes and genomes (KEGG) automatic annotation server (KAAS). All the CDS were compared against the KEGG database using BLASTX with threshold bit-score value of 60 (default). The KEGG orthology (KO) assignment reconstructions were performed in KAAS Ver. 1.6 (http://www.genome.jp/tools/kaas/) with default parameters. KAAS provides functional annotation of genes by BLAST comparison against the manually curated KEGG genes database. The results contain KEGG Orthology (KO) assignments and KEGG pathways.

#### Differential gene expression analysis

The high quality reads for each sample was mapped on their respective set of CDS using CLC Genomic workbench to get the read counts which were used in DESeq 1 [59] to obtain significantly DE genes between young and mature leaf samples. Common hit accessions based on BLAST against NCBI ‘green plant database (txid 33090)’ considering E-value1e-6 were considered for differential gene expression analysis. A complete linkage hierarchical cluster analysis was performed on top 100 differentially expressed genes using Multiple Experiment Viewer (MEV v4.8.1). Levels of expression were represented as log2 ratio of transcript abundance between young and mature leaf samples. Differentially expressed gene identified in young and mature leaf samples were analyzed by hierarchical clustering. A heat map was constructed using the log-transformed and normalized value of genes based on Pearson uncentered correlation distance as well as based on complete linkage method.

#### Simple sequence repeat (SSR) identification, primer designing and validation

For identification of SSRs, all the transcript contigs were searched with Perl script MISA (Microsatellite Searching Tool) (http://pgrc.ipk-gatersleben.de/misa/). The sequences were initially processed and mined for SSR motifs (dimers to hexamers) with a length of 12 bp and above for di-, tri-, tetra, and hexa-nucleotide repeats, and 15 bp and above for penta-nucleotide repeats, using a program MISA (MIcroSAtellite) written in the Perl 5 script language that locates microsatellite patterns in FASTA formatted sequence files and reports the GenBank ID, microsatellite motifs (dimers to hexamers), number of repeats and sequence coordinates for each microsatellite. The rational for choosing the small cutoff value was that the SSRs are often disrupted by single base substitutions [60]. SSRs having a flanking region of 150bp were retained from all the identified SSRs. Microsatellites were classified into class I (20 nucleotides), class II (12–20 nucleotides) and stochastic markers (class III, repeat length of 6–12 nucleotides) based on the length of the microsatellite motifs [61]. SSRs with a motif length of 20 bp and above were selected for designing primers. Primer pairs flanking SSRs were selected using Primer3 software (http://frodo.wi.mit.edu/primer3/). The key parameters set for primer design were as follows: primer length 18–24 bp with 20 bp as the optimum; PCR product size 100–300 bp; optimum annealing temperature 50^°^C; GC content 35–60% with 50% as the optimum. The canonical name proposed for designating markers includes function [unknown (X)], lab designator (DMAPR, Anand (da)], species [*Cassia angustifolia* (ca)], type of marker [EST-microsatellite (em)] and serial no. of marker. Hence, the markers developed in this study were named ‘‘Xdacaem’ for markers. The primers were synthesized by Xcelris Genomics Ltd, Ahmedabad, India. The genomic DNA of 48 germplasm accessions of senna were extracted using the CTAB method. PCR reactions were set up in a 15µl reaction mixture in 96-well PCR plates (Axygen, PCR-96-HSC). Each PCR reaction mixture contained 2–4 pmol of primer, 1–4 mM MgCl_2_, 0.1–0.2 mM dNTP, 0.75 U Taq DNA polymerase and 1.5µl 10x PCR buffer (Sigma–Aldrich, St. Louis, MO, USA) and 30–50 ng of genomic DNA as a template. Temperature cycling was carried out using the S1000 Thermal Cycler (Bio-Rad Laboratories, Philadelphia, PA, USA) and touch-down PCR amplification [62]: one 15-min denaturation cycle, followed by ten cycles of 94^°^C for 10 s, 61^°^C for 20 s (reducing by 1^°^C per cycle) and 72^°^C for 30 s, then by 31 cycles of 94^°^C for 10 s, 54^°^C for 20 s and 72^°^C for 30 s. After completion of the 31 cycles, a final extension of 20 min at 72^°^C was included to minimize the +A overhang [63]. PCR products were separated on 3% agarose (Xcelris Genomics Ltd, Ahmedabad, India) gels.

**Fig 1:**
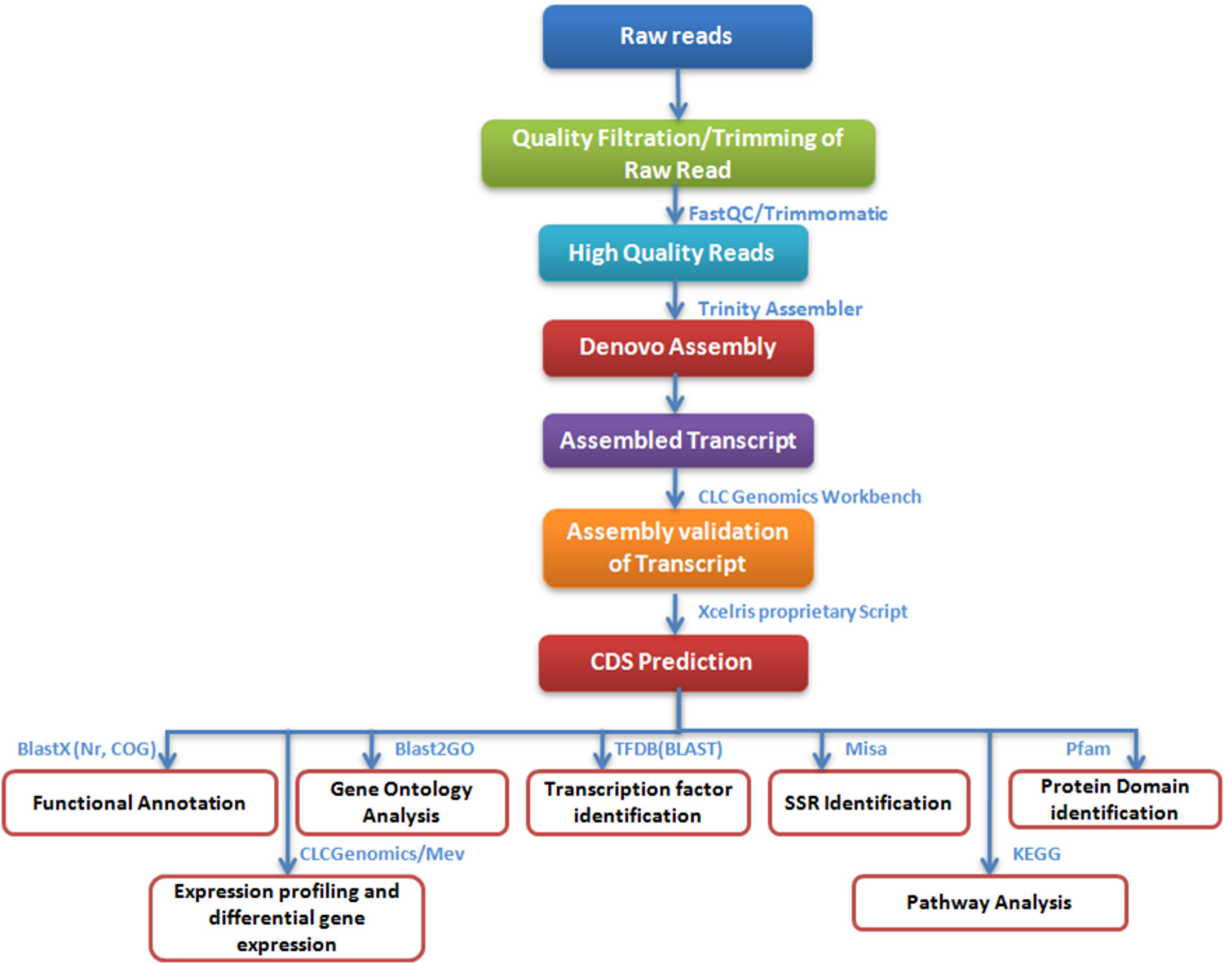
Workflow for Illumina sequencing, de novo assembly, annotation, and other analysis carried out in the leaf transcriptome of *Cassia angustifolia*.

Workflow for Illumina sequencing, *de novo* assembly, annotation, and other analysis carried out in the leaf transcriptome of *Cassia angustifolia* is given in Fig 1.

## Results

### Sequence quality and *de novo* assembly

cDNA libraries prepared from the RNA from the young and mature leaf tissues of *Cassia angustifolia* were sequenced on Illumina Miseq platform and a total of 9128985 and 12897344 raw reads were generated comprising of 2611978510 and 3734013692 nucleotide bases in young and mature leaf libraries, respectively (Table 1). After the quality filtration (mean quality score > = 20) and adaptor trimming using Trimmomatic, the high quality reads were used for *de novo* assembly using Trinity. The raw reads were submitted to the NCBI database and assigned numbers SRS654537 (young) and SRS654538 (mature). High quality sequence of young and matured leaf samples were assembled *de novo* using Trinity RNA-Seq assembler. This Transcriptome Shotgun Assembly project has been deposited at DDBJ/EMBL/GenBank under the accession GCZV00000000 (Young leaf) and GCVR00000000 (mature leaf). The version described in this paper is the first version. Assembled transcript contigs were validated using CLC Bio Genomics workbench by mapping high quality reads back to the assembled transcript contigs. The assembly resulted in a total of 42,230 non-redundant transcripts with N_50_ value of 1239 bp, largest contig length of 7442 bp and the average conting length of 1119 bp in young leaf whereas, it was 37174 non-redundant transcripts with N_50_ value of 1501 bp, largest contig length of 12979 bp and the average contig length of 1467 bp in mature leaf (Table 2). The size distribution of transcripts ranged from <1000 bp to 3,500 bp and above, wherein the maximum number of transcripts (21,083) were in the range of <1000bp followed by 12874 transcripts in the range of 1000–1499 bp in young leaf while in the mature leaf, the maximum number of transcripts (18597) were in the range of 1000 to 1499 bp followed by 9369 transcripts in the range of 1500 to 1999 bp since the number decrease as the transcript length increases (S2 Fig). ATGC composition of the assembled transcripts is given in S3 Fig GC content of C. *angustifolia* transcripts was 42.40% for young leaf and 42.22% for mature leaf with an average of 42.31%.

**Table 1:** Throughput and quality of Illumina sequencing of *Cassia angustifolia* leaf transcriptome.

Sample	Raw data	
Leaf	Young	Mature
Total reads	9128985	12897344
Total nucleotides (bp)	2611978510	3734013692
Mean read length (bp)	143	144
Max read length (bp)	150	150

**Table 2:** Assembly and CDS statistics of Illumina sequencing of *Cassia angustifolia* in the leaf transcriptome.

Assembly statistics	Young leaf	Mature leaf
No. of transcript contigs	42230	37174
Maximum length of transcript contig	7442	12979
N_50_ value	1239	1501
GC%	42.40	42.22
Total transcript contig length (in bases)	47293444	54537750
Average transcript contig length (in bases)	1119	1467
Total Number of Ns	0	0
Transcript contig > 500b	42230	37174
Transcript contig > 1Kb	21123	32587
Transcript contig > 10Kb	0	1
**CDS statistics**		
Total number of CDS	42,230	37,174
Maximum length of CDS (in bases)	5,361	4,146
Minimum length of CDS (in bases)	201	201

### Validation and functional annotation of *Cassia angustifolia* transcripts

Assembled transcript contigs were validated by mapping high quality reads back to the assembled transcript contigs. We observed 91.5% and 85.2% of reads, respectively from the young and mature leaf libraries were mapped to the transcript thereby suggesting that the assembly was highly valid. Due to low expression of certain transcripts, the reads belonging to them might be either partially assembled or left out completely during the assembly process. This leads to a small fraction of reads unused during the assembly process. In our study, 8.5% and 14.8% of the reads respectively, in young and mature leaf libraries did not align back to the transcript reference sequences. We used ORF-Predictor, to identify the coding DNA sequences (CDS) from assembled transcript contigs. A total of 42,230 and 37,174 CDS were obtained for young and mature leaf samples, respectively (Table 2) with a maximum CDS length of 5361 bp in young and 4146 bp in mature leaf. The size distribution CDS according to their length was computed wherein the maximum number of CDS were in range of 1000 and above bp (8977 CDS) which was followed by 400 to 499 (5380 CDS) in the transcriptome of young leaf. While in the mature leaf transcriptome the maximum number of CDS were in range of 1000 and above bp (13394 CDS) which was followed by 700 to 799 (4031 CDS) in the transcriptome (S4 Fig). The functional annotation was performed by aligning those CDS to NCBI ‘green plant database (txid 33090)’ using BLASTX with an E-value threshold of 1e^-06^. We obtained BLAST hits of 40138 (95.0%) and 36349 (97.7%) CDS in young and matured leaf samples, respectively (Table 3). A large number *C*. *angustifolia* CDS showed significant similarity with the *Glycine max* (38.0 and 41.0%, respectively in young and mature leaves) which was followed by *Phaseolus vulgaris* (15.0 and 16.0% respectively in young and mature leaf) and *Cicer arietinum* (13.0 and 14.0% respectively in young and mature leaves) (S5 Fig).

**Table 3:** Distribution of blast results of CDS in the C*assia*. *angustifolia* leaf transcriptome.

Leaf	No. of CDS	No. of CDS with Blast hits	No. of CDS with noBlast hits	GO distribution of BLAST hits		
				Biological processes	Molecular functions	Cellular component
**Young**	42,230	40,138	2,092	19,811	20,578	12,889
**Mature**	37,174	36,349	825	18,156	18,823	11,487

**Fig 2:**
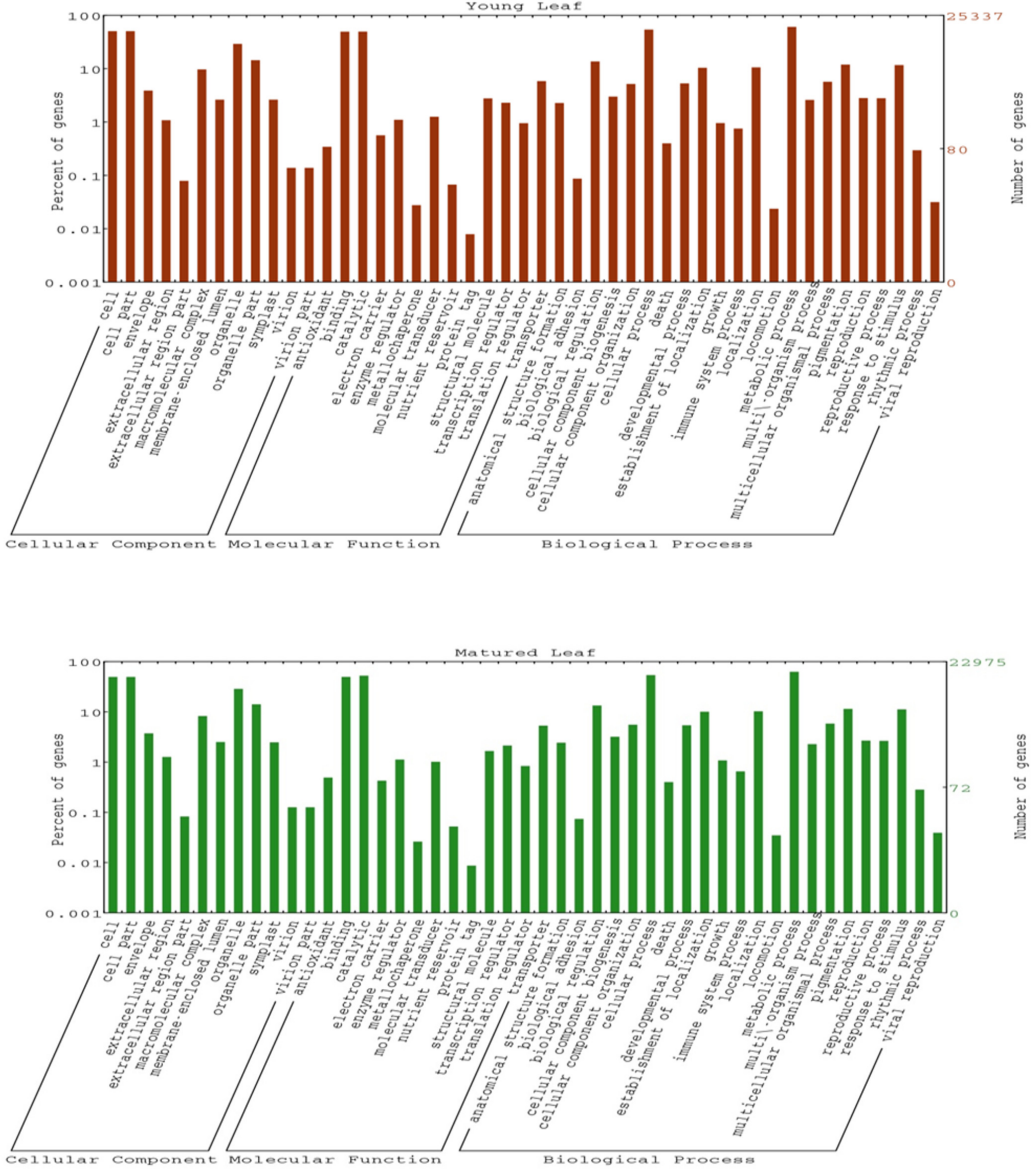
GO Classification in A) young and B) mature leaf transcriptome of *Cassia angustifolia*. *Cassia angustifolia* CDS were searched against the non-redundent protein sequences available in the Uni-ProtKB/SwisProt database using BLASTX with E value threshold of 1e^-06^ in order to assign putative function. Out of 42,230 and 37,174 CDS in young and mature leaf respectively, 29,944 (70.9%) CDS in young and 28,099 (75.5%) CDS in mature leaf transcriptome showed significant hits to the Uni-ProtKB/SwisProt data set thereby showing overall gene conservation. In addition, many *C*. *angustifolia* transcripts showed homology to uncharacterised proteins annotated as unknown, hypothetical and expressed proteins.

Gene ontology (GO) assignments were used to classify the functions of the predicted CDS. The GO mapping also provides ontology of defined terms representing gene product properties which were grouped into three main domains: Biological process, Molecular function, and Cellular component. GO terms were assigned for 25,337 and 22,975 annotated CDS in young and matured leaves, respectively (Table 3 and S6 Fig). As one GO term can be assigned to multiple CDS and the single CDS can have multiple GO terms, a total number of 53,278 and 48,466 GO terms were enriched for the annotated CDS in young and mature leaves, respectively. In young leaf, we have obtained 19,811 terms in biological process, 20,578 terms in molecular functions and 12,889 terms in cellular component (Table 3; S6 Fig). For mature leaf, 18,156 terms were mapped into biological process, 18,823 terms were mapped into molecular functions, and 11,487 terms were mapped into cellular component (Table 3; S6 Fig). The WEGO plots were plotted based on GO hits and CDS were categorized into 45 functional groups from WEGO analysis (Fig 2). In the biological process category, metabolic process (GO:0008152) (young 15391, 60.7% and mature 14395, 62.7%) followed by cellular process (GO:0009987) (young 13691, 54.0% and mature 12381, 53.9%) were prominent in young and mature leaves, suggesting these CDS might be involved in some important metabolic activities in senna. In the molecular function category, “catalytic activity” (GO: 0003824) (young 12555, 49.6% and mature 12049, 52.4%) represented most abundant term, followed by “binding activity” (GO: 0005488) (young 12513, 49.4% and mature 11394, 49.6%). Extremely low percentage of genes were classified in terms of “protein tag” (GO: 0031386), “locomotion” (GO: 0040011), “metallochaperone” (GO: 0016530) and “viral reproduction” (GO: 0016032) in young as well as mature leaf transcriptomes. Under the cellular component category, highest number of CDS was associated with “cell” (GO: 0005623) (young 12790, 50.5% and mature 11377, 49.5%) and “cell part” (GO: 0044464) (young 12790, 50.5% and mature 11377, 49.5%) followed by organelle (GO: 0043226) (young 7382, 29.1% and mature 6580, 28.6%) in young and mature leaf. Both the libraries showed similar type of distribution pattern of CDS under different GO terms.

**Fig 3:**
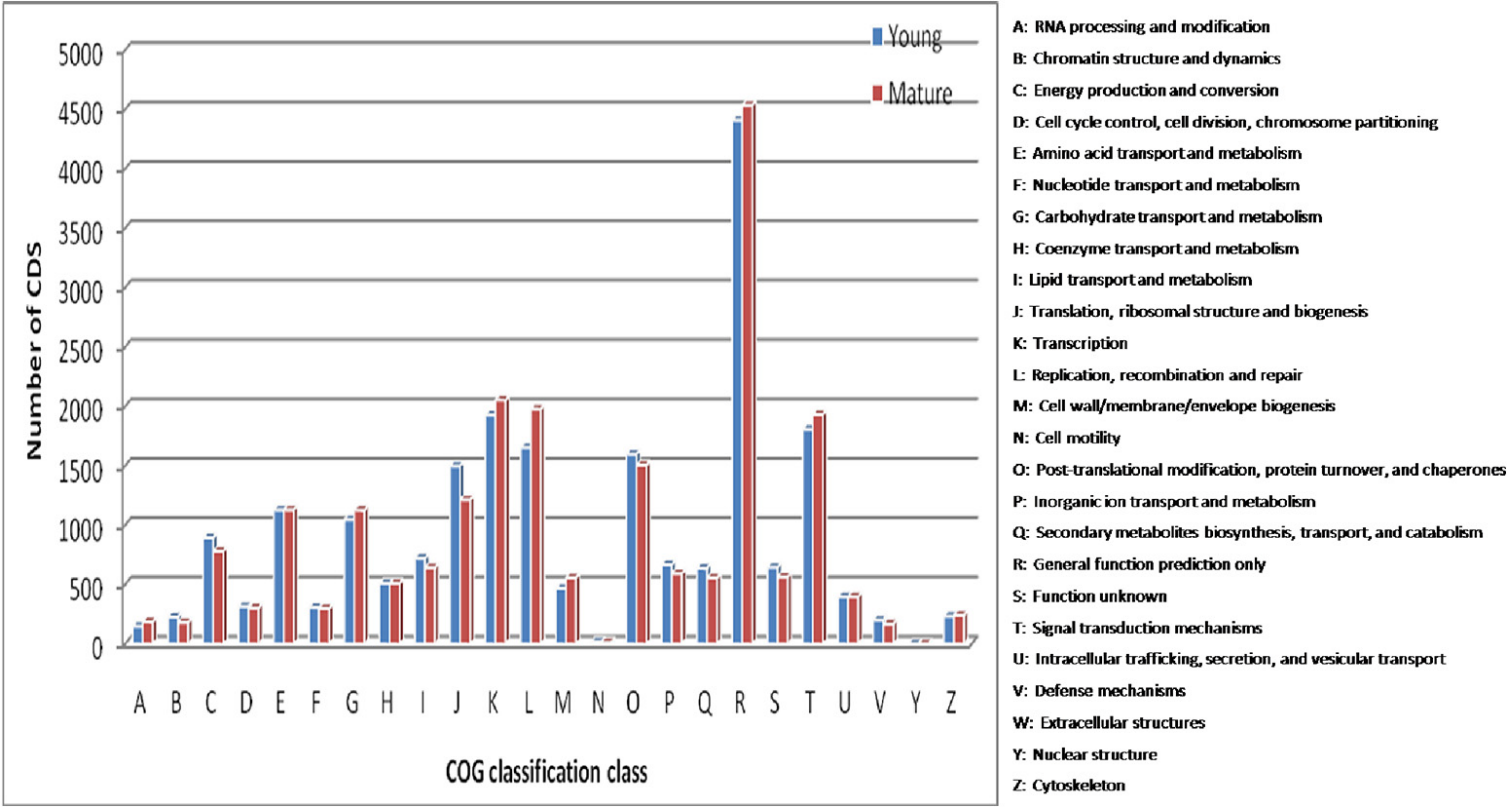
Clusters of orthologous groups (COG) functional classification of CDS in the young and mature leaf transcriptome of *Cassia angustifolia*.

To further predict the function of the CDS, all 42,230 and 37,174 CDS of young and mature leaves, respectively were subjected to classification into different protein families based on Clusters of Orthologus Groups (COG) of protein databases. Overall 15,592 and 15,215 CDS of young and mature leaves, respectively showed significant homology and assigned to the appropriate COG clusters. The COG annotated putative proteins were distributed functionally into at least 24 protein families (Fig 3), of which the cluster for “general function prediction” represented the largest group (4411 and 4534), followed by “transcription” (1927 and 2054), “signal transduction” (1806 and 1932), “replication and repair” (1653 and 1976) and “post translational modification, protein turnover (1598 and 1505) in young and mature leaf samples. The least represented groups include “cell motility” (16 and 12) and “nuclear structure” (3 and 3) in young and mature leaves, respectively.

**Fig 4:**
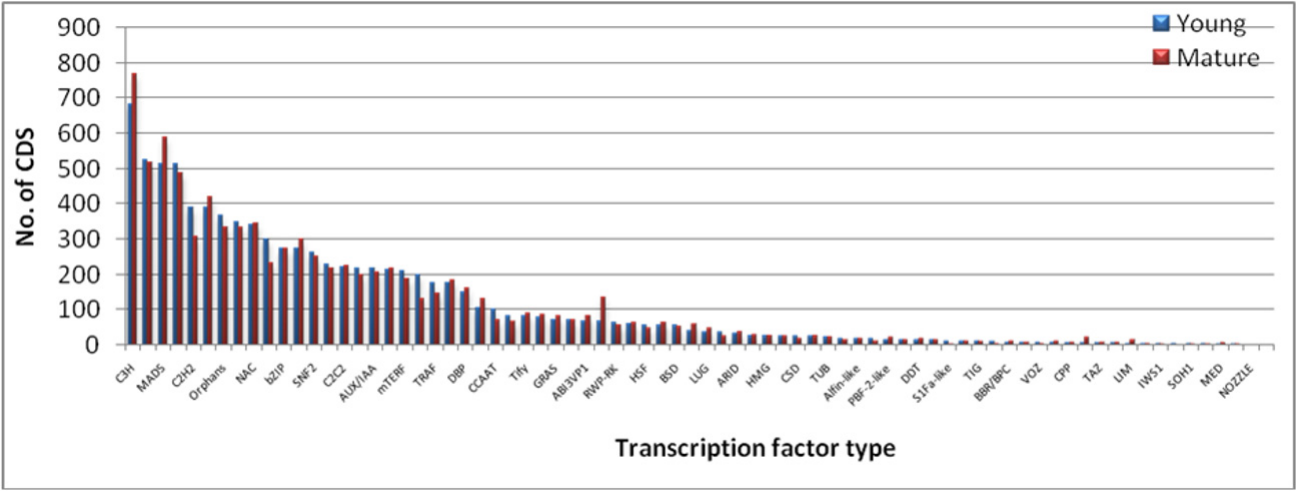
Distribution of the different classes of Transcription factors in the leaf transcriptome of *Cassia angustifolia*.

Transcription factors (TFs) affect metabolic flux by regulating gene expression of particular gene encoding enzymes involved in the biosynthetic pathway and their information would be helpful in manipulating metabolic pathways in plants. In this study, BLASTX with threshold E value of ≤ 1E^-05^ was performed to search against the known Plant Transcription Factor database (http://plntfdb.bio.uni-potsdam.de/v3.0/blastform.php) using the CDS of young and mature leaf separately. Out of 42,230 CDS in young leaf, 8761 (20.7%) CDS were identified to be TFs that belonged to 75 known TF families (Fig 4). Similarly, out of 37,174 CDS in mature leaf, 8715 (23.4%) CDS were identified to be TFs that belonged to 76 known TF families. In the most abundant families 681, 523, 515 and 513 CDS in young, 767, 518, 587 and 489 CDS in mature leaf were annotated to C3H, bHLH, MADS, and MYB families, respectively.

**Fig 5:**
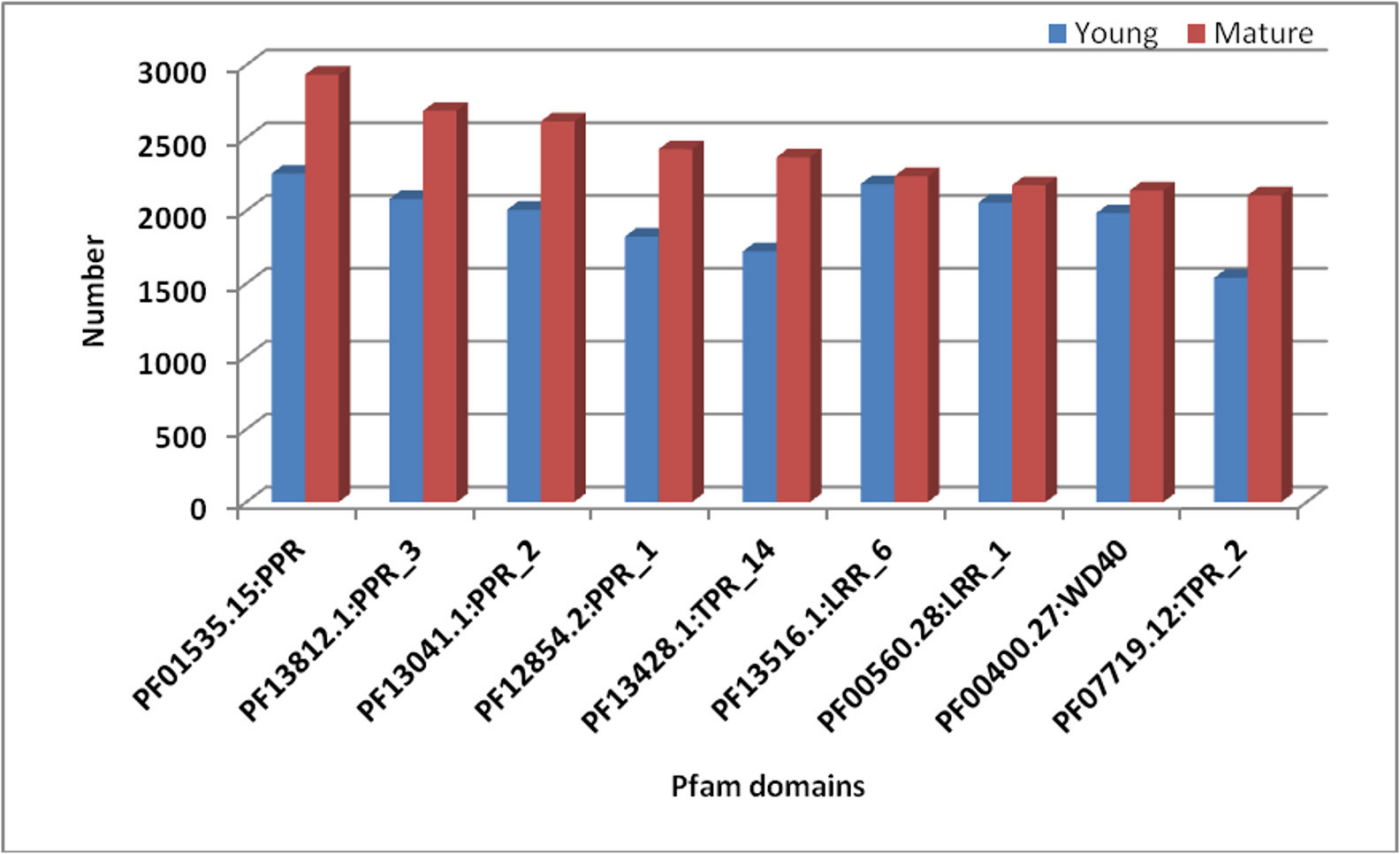
Top 10 Pfam domains represented in InterProScan transcript annotations of the *Cassia angustifolia* leaf Transcriptome.

We used InterProscan to see protein similarity at domain level, where the proteins have little similarity at sequence level but might share conserved structural domains. In total, 34,031 and 32,077 transcripts were annotated against the Pfam domains (Fig 5). Pentatricopeptide repeat (PPR) (PF01535.15) domain represented the most (2255 and 2935 transcripts in young and mature leaves, respectively) which was followed by PPR_3 (PF13812.1) (2081 and 2686 transcripts in young and mature leaves, respectively), PPR_2 (PF13041.1) (2007 and 2614 transcripts in young and mature leaves, respectively) and PPR_1 (PF12854.2) (1823 and 2422 transcripts in young and mature leaves, respectively). Other domains frequently represented in young and mature leaf libraries include TPR_14 (PF13428.1) (1721 and 2367), LRR_6 (PF13516.1) (2183 and 2237), LRR_1 (PF00560.28) (2056 and 2175) and WD40 (PF00400.27) (1983 and 2139) in the transcripts indicating strong signal transduction mechanisms.

**Fig 6:**
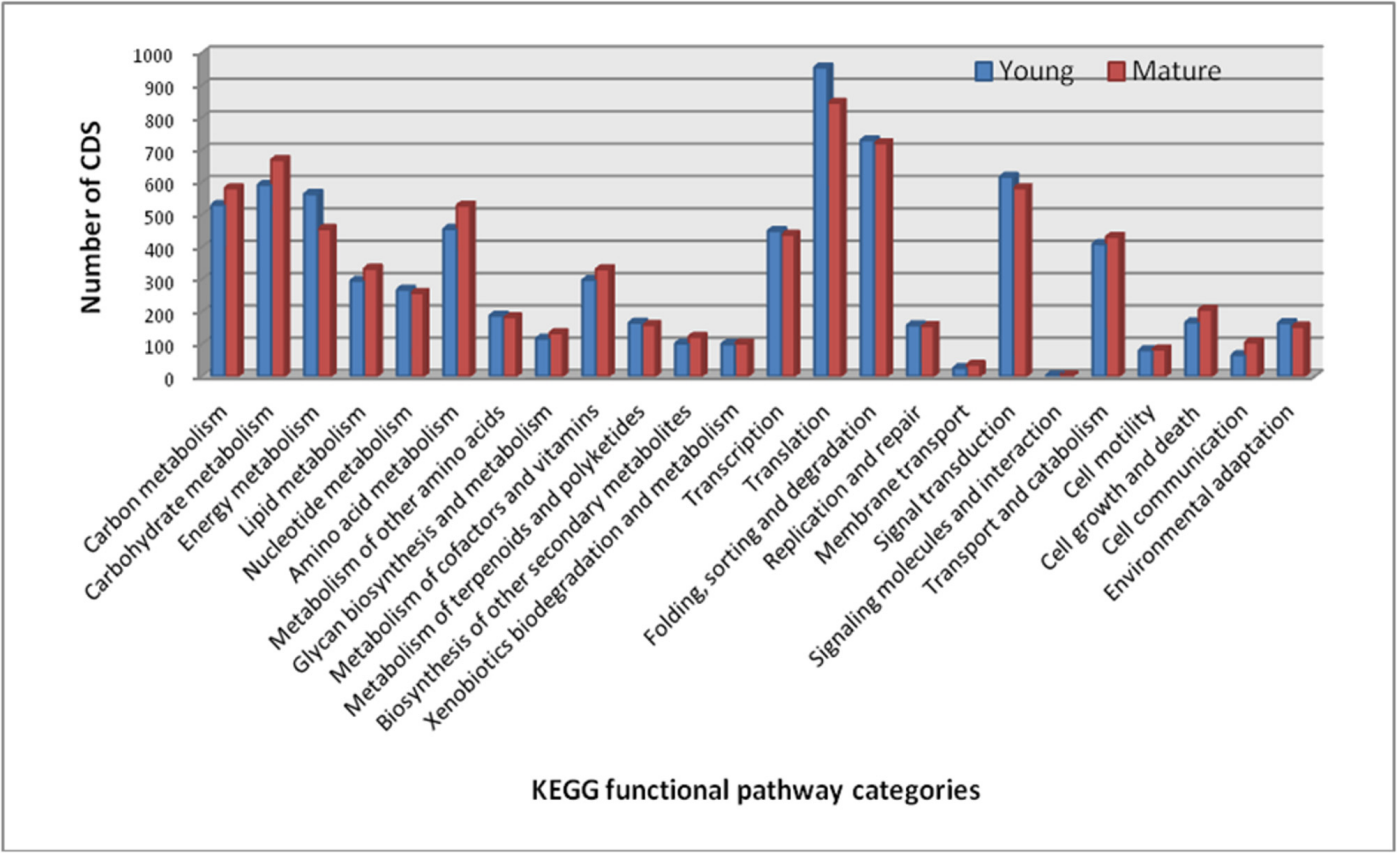
KEGG pathway analysis of CDS in the young and mature leaf transcriptome of *Cassia angustifolia*.

Pathway based analysis can help us further understand the biological significance of genes. The Koyto Encyclopedia of Genes and Genomes (KEGG) pathway database contains systematic analysis of inner-cell metabolic pathways and functions of gene products, which aid in studying the complex biological behavior of genes. Ortholog assignment and mapping of the CDS to the biological pathways were performed using KEGG automatic annotation server (KAAS). All the CDS were compared against the KEGG database using BLASTX with threshold bit-score value of 60 (default). A total of 7,504 and 7,618 CDS were enriched in 24 different functional KASS pathway categories in young and mature leaf, respectively (Fig 6). The mapped CDS represented metabolic pathways of major biomolecules such as carbon, carbohydrates, lipids, nucleotides, amino acids, glycans, cofactors, vitamins, terpenoids, polyketides, and others. The mapped CDS also represented the genes involved in genetic information processing, environmental information processing, cellular processes, and organizational systems. In total, all CDS from young and mature leaf were assigned to 191 KEGG pathways (S1 Table and S2 Table). In young leaf, the pathways with most representation by the CDS were translation (955) followed by folding, sorting and degradation (730), and signal transduction (617). While in mature leaf, translation (846) followed by folding, sorting and degradation (721), and carbohydrate metabolism (669) were the most represented pathways by the CDS. The least represented pathways include ‘signal molecules and interaction’, and membrane transport. Interestingly, 166 and 159 CDS, from young and mature leaf libraries, respectively, were found to be involved in metabolism of terpenoids and polyketides. Within this category, the cluster for ‘Terpenoid backbone biosynthesis [PATH:ko00900]’ represented the largest cluster with 58 and 66 CDS in young and mature leaf libraries, respectively. Similarly, there were 102 and 122 CDS from young and mature leaves, respectively, were found to be involved in the biosynthesis of other secondary metabolites. The ‘Phenylpropanoid biosynthesis [PATH: ko00940]’ cluster represented the largest group with 48 and 60 CDS in young and mature leaf libraries, respectively.

### Genes involved in anthraquinones biosynthesis

**Fig 7:**
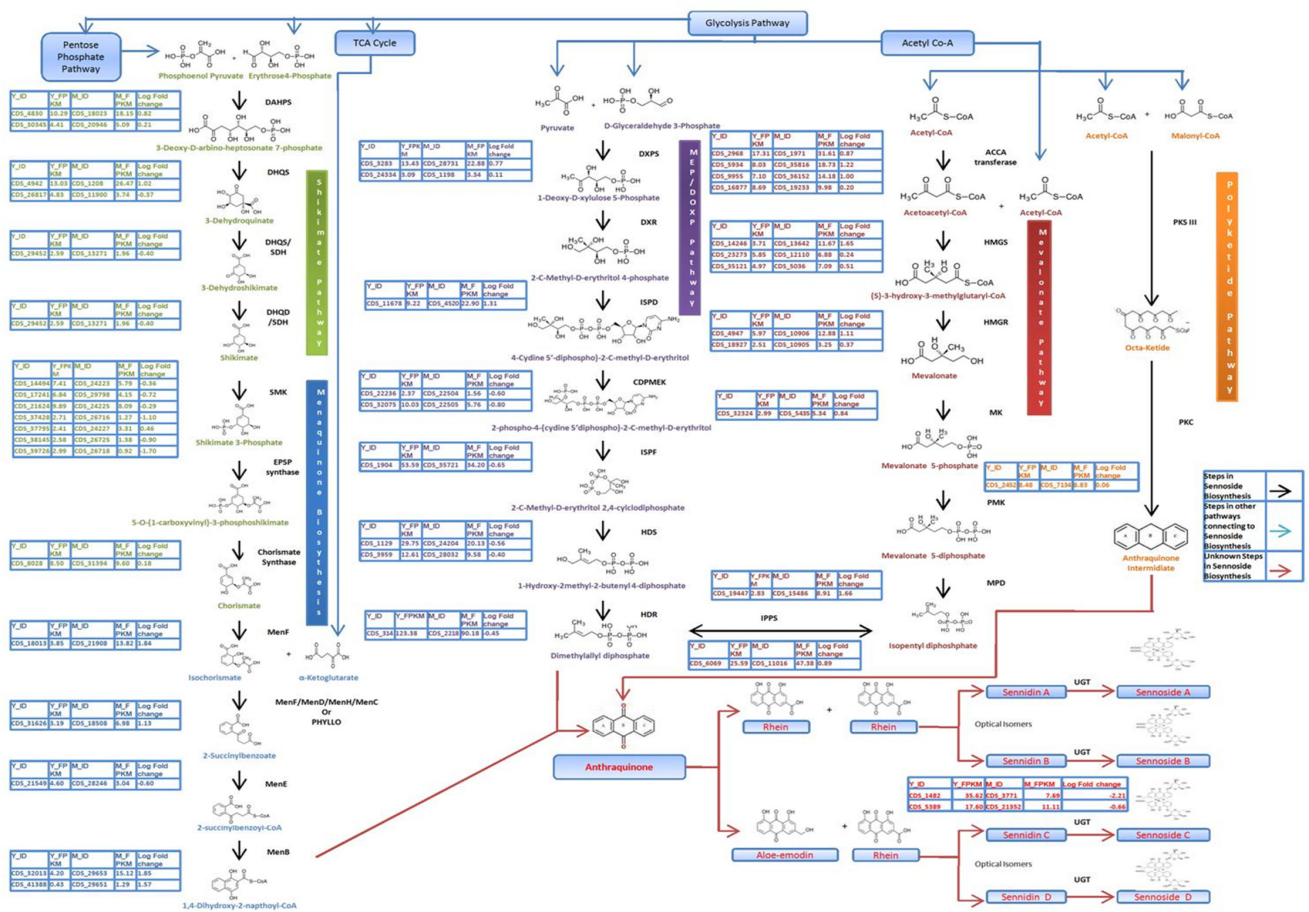
Mapping of the genes expressing differentially in young vs mature leaf on the putative sennoside biosynthetic pathway in *Cassia angustifolia*. DAHPS:3-deoxy-7-phosphoheptulonate synthase[EC:2.5.1.54], DHQS: 3-dehydroquinate synthase[EC:4.2.3.4],DHQS/SDH: 3-dehydroquinate dehydratase / shikimate dehydrogenase[EC:4.2.1.10 1.1.1.25], SMK: shikimate kinase[EC:2.7.1.71], EPSP Synthase: 3-phosphoshikimate 1-carboxyvinyltransferase/enolpyruvylshikimate phosphate synthase [EC:2.5.1.19], chorismate synthase [EC:4.2.3.5] menF: isochorismate synthase [EC:5.4.4.2], menF/menD/menH/menC or PHYLLO: isochorismate synthase / 2-succinyl-5-enolpyruvyl-6-hydroxy-3-cyclohexene-1-carboxylate synthase / 2-succinyl-6-hydroxy-2,4-cyclohexadiene-1-carboxylate synthase / O-succinylbenzoate synthase[EC:5.4.4.2 2.2.1.9 4.2.99.20 4.2.1.113], menE: Succinylbenzoic acid-CoA ligase/acyl-activating enzyme 14 [EC:6.2.1.26], menB: naphthoate synthase[EC:4.1.3.36], DXPS: 1-deoxy-D-xylulose-5-phosphate synthase[EC:2.2.1.7], DXR: 1-deoxy-D-xylulose-5-phosphate[EC:1.1.1.267], ISPD: 2-C-methyl-D-erythritol 4-phosphate cytidylyltransferase [EC:2.7.7.60], CDPMEK:4-diphosphocytidyl-2-C-methyl-D-erythritol kinase[EC:2.7.1.148], ISPF: 2-C-methyl-D-erythritol 2,4-cyclodiphosphate synthase[EC:4.6.1.12], HDS: (E)-4-hydroxy-3-methylbut-2-enyl-diphosphate synthase[EC:1.17.7.1], HDR: 4-hydroxy-3-methylbut-2-enyl diphosphate reductase[EC:1.17.1.2], ACCP Transferase: acetyl-CoA C-acetyltransferase [EC:2.3.1.9], HMGS:hydroxymethylglutaryl-CoA synthase [EC:2.3.3.10], HMGR: hydroxymethylglutaryl-CoA reductase (NADPH) [EC:1.1.1.34], MK: mevalonate kinase[EC:2.7.1.36], PMK: phosphomevalonate kinase [EC:2.7.4.2], MPD: diphosphomevalonate decarboxylase[EC:4.1.1.33], IPPS: isopentenyl-diphosphate delta-isomerase[EC:5.3.3.2], PKS: Polyketide Synthase, PKC:Polyketide Cyclase, UGT:UDP-Glucosyl Transferase; Y_ID:Young leaf CDS ID number for enzyme, M_ID:Mature leaf CDS ID number for enzyme, Y_FPKM: Young leaf CDS FPKM value, M_FPKM: Mature leaf CDS FPKM value, Log Fold Change: Log of change in folds of expression of CDS in young compared to matured leaf transcripts.

The biosynthesis of anthraquinone shares isochorismate pathway with phenylpropanoid and shares MVA/MEP with sterol and (or) terpenoids (Fig 7). In our study there were 31 CDS in young and 29 CDS in mature leaf libraries for six enzymes involved in the Mevalonate pathway leading to production of precursor dimethylallyl diphospahate (Table 4). Dimethylallyl diphospahate is also produced through non-mevalonate pathway (MEP pathway), there were 38 and 34 CDS in young and mature leaves, respectively, for eight enzymes involved in the MEP pathway. Biosynthesis of anthraquinone shares isochorismate pathway or skimate pathway leading to production of the precursor chrorismate which intern forms a substrate for the production of 1, 4-Dihybroxy-2-napthoyl-Co-A, a precursor for anthraquinone production in the menoquinone pathway. We have identified 64 and 78 CDS encoding for seven enzymes in the shikimate pathway in young and mature leaf libraries, respectively. Similarly, for four enzymes in menoquinone pathway, we have identified 14 CDS in young and 22 CDS in mature leaf libraries. Anthraquinone are also known to be produced from acetyl co-A and melonyl co-A through polyketide pathway in plants. Polyketide synthease III is an important enzyme involved in the polyketide pathway. In our study there were two CDS each in young and mature leaves encoding for enzymes involved in polyketide pathway. Bioactive natural products are frequently glycosylated with saccharide chains of different length, in which the sugars contribute to specific interactions with the biological target. In general, glycosylation takes place at the end of secondary metabolites biosynthesis and results in both increased stability and water solubility of secondary metabolites. In nature, glycosylation is normally carried out by UDP-glycosyltransferase, and the natural product carrying a hydroxyl group being the site for glycosylation. Glucosylation (addition of glucose) of hydroxyl group at C_8_ moiety of sennosides is essential for the activity. In our study, there were seven CDS in young and four CDS in mature leaf encoding UDP-glucosyltransferase (Table 4 and Fig 7). Transcriptome sequencing studies offer a wealth of genes sequences involved in various secondary metabolite biosynthesis processes, more specifically those of Cytochrome P450 (CYP or P450). CYPs are membrane bound hemoproteins involved in array of pathways in primary and secondary metabolism in plants. Most of the oxidative reactions, including hydroxylations, epoxidation, dealkylation, dehydration and carbon-carbon bond cleavage of metabolites are catalyzed by CYP group of enzymes. Therefore, it would be pertinent to study CYPs in the leaf transcriptome of *C*. *angustifolia*. We found 177 and 121, four and three and eight and nine CDS respectively, in young and mature leaves encoding for CYP450, CYP450 monooxygenase and NADPH-CYP450 reductase enzymes. These CYPs might be involved in sennoside biosynthesis in senna possibly in the formation of anthraquinone from octaketide backbone.

**Table 4:** Putative genes involved in sennoside biosynthesis identified in the leaf transcriptome of *Cassia angustifolia*.

Pathway	Gene	Symbol	CDS in young leaf	CDS in mature leaf
Mevalonate pathway	Acetyl-CoA C-acetyltransferase (EC 2.3.1.9)	ACAT	12	10
	Hydroxymethylglutaryl-CoA synthase (EC 2.3.3.10)	HMGS	7	8
	Hydroxymethylglutaryl-CoA reductase (EC 1.1.1.34)	HMGR	3	4
	Mevalonate kinase (EC 2.7.1.36)	MK	2	3
	Phosphomevalonate kinase (EC 2.7.4.2)	PMK	2	2
	Diphosphomevalonate decarboxylase (EC 4.1.1.33)	MVD	5	2
MEP pathway	1-deoxy-D-xylulose-5-phosphate synthase (EC 2.2.1.7)	DXPS	10	7
	1-deoxy-D-xylulose-5-phosphate reductoisomerase(EC 1.1.1.267)	DXR	6	6
	2-C-methyl-D-erythritol 4-phosphate cytidylyltransferase (EC 2.7.7.60)	ISPD	5	2
	4-diphosphocytidyl-2-C-methyl-D-erythritol kinase(EC 2.7.1.148)	CDPMEK	4	7
	2-C-methyl-D-erythritol 2,4-cyclodiphosphate Synthase (EC 4.6.1.12)	ISPF	2	2
	(E)-4-hydroxy-3-methylbut-2-enyl-diphosphate synthase (EC 1.17.7.1)	HDS	4	5
	4-hydroxy-3-methylbut-2-enyl diphosphate reductase (EC 1.17.1.2)	HDR	2	2
	Isopentenyl-diphosphate delta-isomerase (EC 5.3.3.2)	IPP	5	3
Shikimate pathway	3-deoxy-7-phosphoheptulonate synthase (EC:2.5.1.54)	DAHPS	7	8
	3-dehydroquinate synthase (EC:4.2.3.4)	DHQS	5	8
	3-dehydroquinate dehydratase / shikimate dehydrogenase (EC:4.2.1.10 1.1.1.25)	SDH	9	6
	Shikimate kinase (EC:2.7.1.71)	SMK	37	51
	3-phosphoshikimate 1-carboxyvinyltransferase (EC:2.5.1.19)	EPSP synthase	2	2
	Chorismate synthase (EC:4.2.3.5)	CS	4	3
Menaqunone pathway	Menaquinone-specific isochorismate synthase (EC:5.4.4.2)	menF	4	3
	Isochorismate synthase / 2-succinyl-5-enolpyruvyl-6-hydroxy-3-cyclohexene-1-carboxylate synthase / 2-succinyl-6-hydroxy-2,4-cyclohexadiene-1-carboxylate synthase / O-succinylbenzoate synthase (EC:5.4.4.2 2.2.1.9 4.2.99.20 4.2.1.113)	menF/menD/ menH/menC or PHYLLO	2	3
	Acyl-activating enzyme 14 (EC:6.2.1.26)	menE	4	12
	Naphthoate synthase (EC:4.1.3.36)	menB	4	7
Polyketide pathway	Polyketide synthases III	PKSIII	1	0
	Polyketide cyclase/dehydratase	PKC	1	2
	Polyketide hydrolase	PH	0	0
Glycosylation	UDP-Glucosyl Transferase	UGT	7	4
CYPs	Cytochrome P450 (EC: 1.14)	CYP	177	121
	Cytrochrome P450 Monooxygenase (EC: 1.14.13)	-	4	3
	NADPH-cytochrome P450 reductase (EC 1.6.2.4)	-	8	9

### Over view of the differentially expressed genes in the young and mature leaf transcriptome of senna

**Fig 8:**
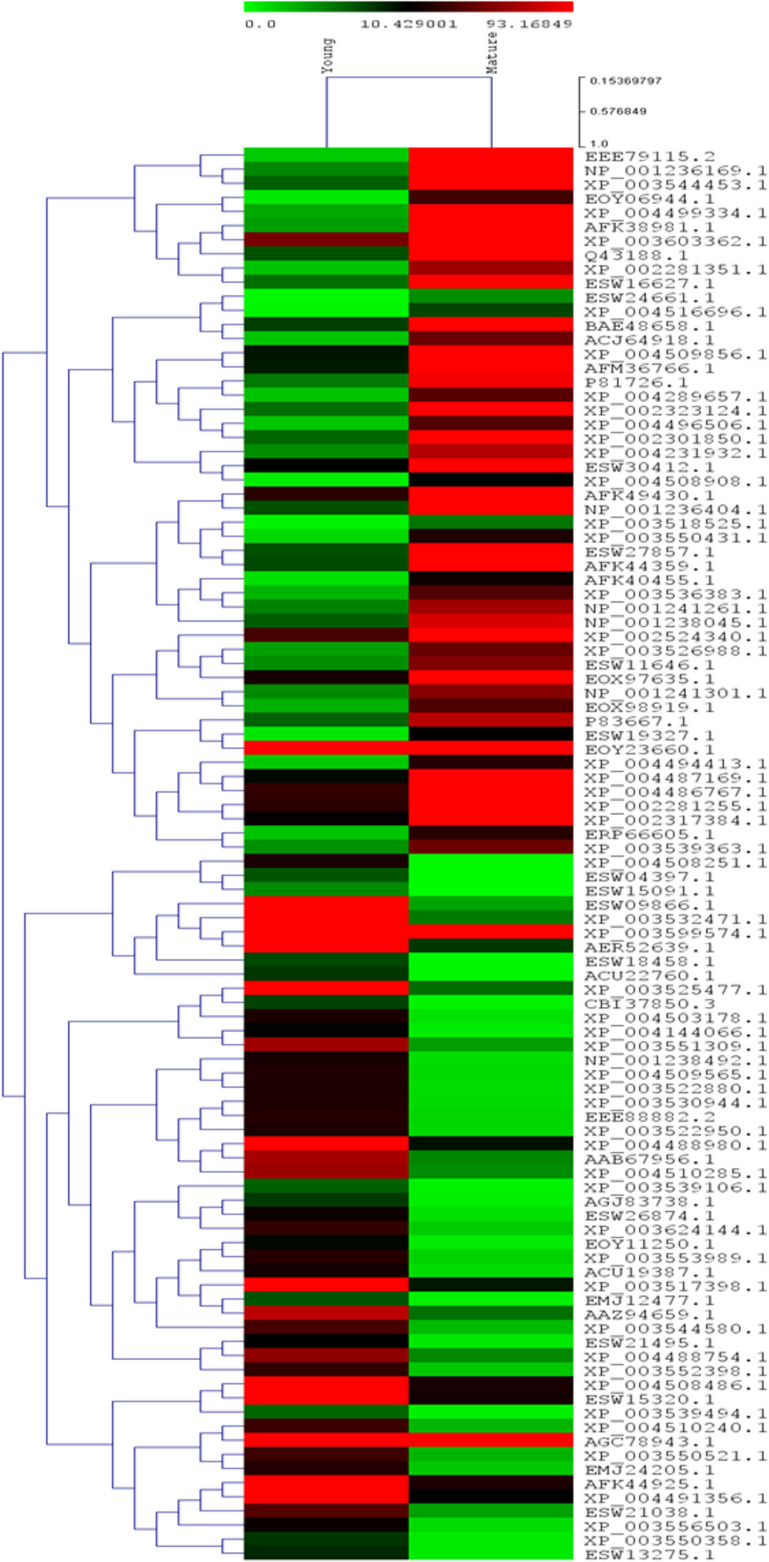
Heat map of top 100 differentially expressed genes in young and mature leaf transcriptome of *Cassia angustifolia*.

**Fig 9:**
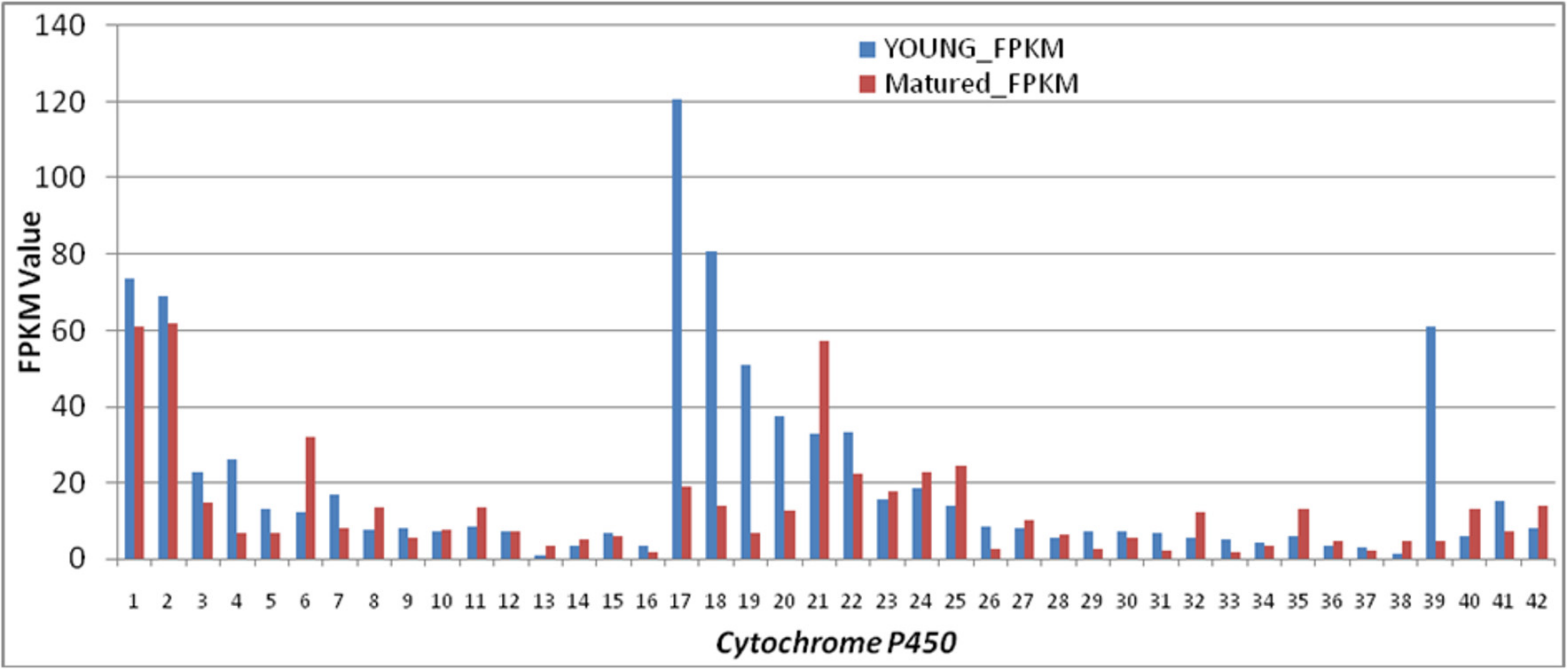
FPKM values based gene expression of annotated cytochrome P450s (CYPs) in leaf transctiptome of *Cassia angustifolia*.

Differential gene expression profile between the young and mature leaf transcripts was created using multiple experiment viewer (MEV v4.8.1) to identify genes with differential expression level in the young leaf compared to mature leaf (as control), initially we used the FPKM method (fragments per kilobase of transcript per million fragments mapped) to calculate the expression level of the CDS. Differentially expressed gene identified in control and experimental conditions were analyzed by hierarchical clustering. A heat map was constructed using the log-transformed and normalized value of genes based on Pearson uncentered correlation distance as well as based on complete linkage method (Fig 8). Based on the common hit accession of functionally annotated CDS in young and mature leaf CDS, a total of 10,763 CDS expressing in both young and mature leaf libraries of which a total of 333 (3.09%) CDS were down-regulated in young leaf compared to mature leaf whereas 2,343 (21.7%) CDS were up-regulated in young leaf compared to mature leaf with the log 2 fold change value of greater than zero (S3 Table). Gene ontology (GO) enrichment analysis was performed with the 2,343 CDS up-regulated in young leaf compared to mature leaf (S4 Table). The GO terms ‘metabolic process’ (GO:0008152) was mostly significantly enriched, followed by ‘translation’ (GO:0006412), ‘oxidation-reduction process’ (GO:0055114), ‘protein phosphorylation’ (GO:0006468) and ‘proteolysis’ (GO:0006508). The BLASTX search was performed with the 2,343 CDS up-regulated in young leaf compared to mature leaf in DGE data, we obtained number of them to be functionally involved in the anthraquinone biosynthetic pathway, such as three CDS (CDS_2968, CDS_5934 and CDS_9955) encoding ACAT and one CDS each encoding HMGS (CDS_14246), HMGR (CDS_4947), MVD (CDS_19447), DXPS (CDS_3283), ISPD (CDS_11678), IPP (CDS_6069), DAHPS (CDS_4830), DHQS (CDS_4942), menF (CDS_18013) and menB (CDS_32013) were up-regulated in young leaf compared to mature leaf however with varying log2 fold change (Table 5). BLAST search also identified 42 CDS encoding for CYPs found differentially expressing (Fig 9; S5 Table) of which 19 CYPs were up-regulated in young leaf compared to mature leaf suggests these to be possible candidates associated with sennoside biosynthesis.

**Table 5:** Differential expression of genes involved in the sennoside biosynthesis in the *Cassia angustifolia* leaf transcriptome.

Pathway	Gene	Symbol	Common Hit Accession*	Young leaf	FPKM^#^	Mature leaf	FPKM^#^	FC^$^	P-value	Regulation**	Significance
Mevalonate Pathway	Acetyl-CoA C-acetyltransferase (EC 2.3.1.9)	ACAT	NP_001267690.1	CDS_2968	17.31	CDS_1971	31.61	0.87	0.00	UP	YES
			XP_003545555.1	CDS_5934	8.03	CDS_35816	18.73	1.22	0.01	UP	YES
			ESW25151.1	CDS_9955	7.10	CDS_36152	14.18	1.00	0.03	UP	YES
			XP_002308755.1	CDS_16877	8.69	CDS_19233	9.98	0.20	0.31	UP	NO
	Hydroxymethylglutaryl-CoA synthase (EC 2.3.3.10)	HMGS	XP_003538436.1	CDS_14246	3.71	CDS_13642	11.67	1.65	0.01	UP	YES
			ESW24350.1	CDS_23273	5.85	CDS_12110	6.88	0.24	0.38	UP	NO
			EOY24602.1	CDS_35121	4.97	CDS_5036	7.09	0.51	0.25	UP	NO
	Hydroxymethylglutaryl-CoA reductase (EC 1.1.1.34)	HMGR	XP_002510732.1	CDS_4947	5.97	CDS_10906	12.88	1.11	0.03	UP	YES
			ABF56518.2	CDS_18927	2.51	CDS_10905	3.25	0.37	0.49	UP	NO
	Mevalonate kinase (EC 2.7.1.36)	MK	AFK41769.1	CDS_32324	2.99	CDS_5435	5.34	0.84	0.22	UP	NO
	Phosphomevalonate kinase (EC 2.7.4.2)	PMK	-	-	-	-	-	-	-	-	-
	Diphosphomevalonate decarboxylase (EC 4.1.1.33)	MVD	XP_004497159.1	CDS_19447	2.83	CDS_15486	8.91	1.66	0.03	UP	YES
MEP pathway	1-deoxy-D-xylulose-5-phosphate synthase (EC 2.2.1.7)	DXPS	AAQ84169.1	CDS_3283	13.43	CDS_28731	22.88	0.77	0.01	UP	YES
			XP_003527796.1	CDS_24334	3.09	CDS_1198	3.34	0.11	0.60	UP	NO
	1-deoxy-D-xylulose-5-phosphate reductoisomerase (EC 1.1.1.267)	DXR	-	-	-	-	-	-	-	-	-
	2-C-methyl-D-erythritol 4-phosphate ytidylyltransferase (EC 2.7.7.60)	ISPD	AEM42975.1	CDS_11678	9.22	CDS_4520	22.90	1.31	0.00	UP	YES
	4-diphosphocytidyl-2-C-methyl-D-erythritol kinase(EC 2.7.1.148)	CDPMEK	XP_003543330.1	CDS_22236	2.37	CDS_22504	1.56	-0.60	0.94	DOWN	NO
			XP_004499437.1	CDS_32075	10.03	CDS_22505	5.76	-0.80	0.68	DOWN	NO
	2-C-methyl-D-erythritol 2,4-cyclodiphosphate Synthase (EC 4.6.1.12)	ISPF	NP_001237894.1	CDS_1904	53.59	CDS_35721	34.20	-0.65	0.63	DOWN	NO
	(E)-4-hydroxy-3-methylbut-2-enyl-diphosphate synthase (EC 1.17.7.1)	HDS	XP_003540178.1	CDS_1129	29.75	CDS_24204	20.13	-0.56	0.87	DOWN	NO
			XP_003543457.1	CDS_3959	12.61	CDS_28032	9.58	-0.40	0.87	DOWN	NO
	4-hydroxy-3-methylbut-2-enyl diphosphate reductase (EC 1.17.1.2)	HDR	XP_003537898.1	CDS_314	123.38	CDS_2218	90.18	-0.45	0.82	DOWN	NO
	Isopentenyl-diphosphate delta-isomerase (EC 5.3.3.2)	IPP	EMJ06840.1	CDS_6069	25.59	CDS_11016	47.38	0.89	0.00	UP	YES
Shikimate pathway	3-deoxy-7-phosphoheptulonate synthase (EC:2.5.1.54)3-dehydroquinate synthase (EC:4.2.3.4)	DAHPSDHQS	XP_003545685.1	CDS_4830	10.29	CDS_18023	18.15	0.82	0.02	UP	YES
			XP_002301067.1	CDS_30345	4.41	CDS_20946	5.09	0.21	0.47	UP	NO
			XP_004512152.1	CDS_4942	13.03	CDS_1208	26.47	1.02	0.00	UP	YES
			XP_003554373.1	CDS_26817	4.83	CDS_11900	3.74	-0.37	0.90	DOWN	NO
	3-dehydroquinate dehydratase / shikimate dehydrogenase (EC:4.2.1.10 1.1.1.25)	SDH	AFK48985.1	CDS_29452	2.59	CDS_13271	1.96	-0.40	0.94	DOWN	NO
	Shikimate kinase (EC:2.7.1.71)	SMK	XP_003518273.1	CDS_14494	7.41	CDS_24223	5.79	-0.36	0.86	DOWN	NO
			XP_003517670.1	CDS_17241	6.84	CDS_29798	4.15	-0.72	0.80	DOWN	NO
			AFK43498.1	CDS_21624	9.89	CDS_24225	8.09	-0.29	0.76	DOWN	NO
			EMJ06821.1	CDS_37428	2.71	CDS_26716	1.27	-1.10	0.69	DOWN	NO
			XP_004514688.1	CDS_37795	2.41	CDS_24227	3.31	0.46	0.45	UP	NO
			XP_003531380.1	CDS_38145	2.58	CDS_26725	1.38	-0.90	0.79	DOWN	NO
			AFK43283.1	CDS_39726	2.99	CDS_26718	0.92	-1.70	0.44	DOWN	NO
	3-phosphoshikimate 1-carboxyvinyltransferase (EC:2.5.1.19)	EPSP synthase	-	-	-	-	-	-	-	-	-
	Chorismate synthase (EC:4.2.3.5)	CS	XP_003556230.1	CDS_8028	8.50	CDS_31394	9.60	0.18	0.34	UP	NO
Menaquinone pathway	Menaquinone-specific isochorismate synthase (EC:5.4.4.2)	menF	AHA82396.1	CDS_18013	3.85	CDS_21908	13.82	1.84	0.01	UP	YES
	Isochorismate synthase / 2-succinyl-5-enolpyruvyl-6-hydroxy-3-cyclohexene-1-carboxylate synthase / 2-succinyl-6-hydroxy-2,4-cyclohexadiene-1-carboxylate synthase / O-succinylbenzoate synthase (EC:5.4.4.2 2.2.1.9 4.2.99.20 4.2.1.113)	menF/menD/ menH/menC or PHYLLO	XP_004509573.1	CDS_31626	3.19	CDS_18508	6.98	1.13	0.11	UP	NO
	Acyl-activating enzyme 14 (EC:6.2.1.26)	menE	XP_003535086.1	CDS_21549	4.60	CDS_28246	3.04	-0.60	0.92	DOWN	NO
	Naphthoate synthase (EC:4.1.3.36)	menB	NP_001241269.1	CDS_32013	4.20	CDS_29653	15.12	1.85	0.00	UP	YES
			ESR51586.1	CDS_41388	0.43	CDS_29651	1.29	1.57	0.42	UP	NO
Polyketide pathway	Polyketide synthases III	PKSIII	-	-	-	-	-	-	-	-	-
	Polyketide cyclase/dehydratase	PKC	EOY23837.1	CDS_2452	8.48	CDS_7134	8.83	0.06	0.43	UP	NO
	Polyketide hydrolase	PH	-	-	-	-	-	-	-	-	
Glycosylation	UDP-Glucosyl transferase	UGT	XP_003555960.1	CDS_1482	35.62	CDS_3771	7.69	-2.21	0.00	DOWN	YES
			ESW15045.1	CDS_5389	17.60	CDS_21352	11.11	-0.66	0.76	DOWN	NO

*Common hit accession of the NCBI BLAST annotation,

^#^ FPKM: Fragments Per Kilobase of Exon Per Million Fragments Mapped was calculated using formula, FPKM = 10^9 x C / (N x L), where, C is the number of reads mapped onto the CDS, N the total number of mappable reads in the experiment L the number of base pairs in the CDS,

^$^Fold change (FC) is ratio of Log_2_ (Young/mature). FC value greater than zero were considered up-regulated whereas FC value less than zero were considered down-regulated,

**Up regulated (UP) and Down regulated (Down).

### Microsatellite identification

Simple sequence repeats are short repeat sequences of 2–6 bases which are important molecular markers in a wide range of plant breeding applications. A total of 66,610 SSRs were identified in 31,010 transcripts (Table 6) in the present study using MISA. More than one SSR was found to be in 17,699 (26.5%) transcripts and compound SSRs were observed to be 10,453 (15.6%). The frequency of SSR motifs revealed that hexamers are more frequent (75.23%) followed by trimers (11.92%), dimers (5.75%), tetramer (5.70%), and pentamers (1.39%) (S6 Table). In general, AT rich motifs were found to be more frequent among all types of repeat motifs except for trimers where CG rich motifs were predominantly observed. Among hexamers, motif with 50% AT rich (33.39%) was most common, followed by 66.6% AT rich (31.39%) motifs, whereas 83.3% AT (17.07%), 33.3% AT (12.17%), 100% AT (3.12%), and 16.6% AT (2.65%) and 0% AT (0.21%) rich motifs were found less frequently. Similarly, among the trimers, the motif AAG was most common (30.66%), followed by ATC (16.72%), AGG (12.56%) and AGC (10.60%), whereas the motif ACT was least common (1.61%). However, the dimers, tetramers, or pentamers were found in insignificant numbers (<10%). Of the total number of SSRs studied in the present study, 1752 (2.60%) SSRs were found hypervariable (class I, repeat length of ≥20 bp) of which 725 were dimeric SSRs, 7131 (10.70%) SSRs were potentially variable markers (class II, repeat length of 13–20 bp) of which 1966, 2490, 424, 792, and 1459 respectively were from dimers to hexamers, and remaining were stochastic markers (class III, repeat length of 6–12 bp). The present study, 35 genic SSRs was designed and the PCR amplification of a set of 35 primers reveled amplification in 22 (62%) markers (S7 Table; S7 Fig). These SSRs are highly useful in genetic analysis and molecular breeding of senna.

**Table 6:** Statistics of SSRs identified using MISA in the leaf transcriptome of *Cassia angustifolia*.

SSRs mining	Total
Total number of sequences examined	43413
Total size of examined sequences (bp)	72058285
Total number of identified SSRs	66610
Number of SSR containing sequences	31010
Number of sequences containing more than 1 SSR	17699
Number of SSRs present in compound formation	10453
Frequency per Kb	1.08
Density (bp/Kb)	11.73

### Sennoside content and leaf age of senna

**Fig 10:**
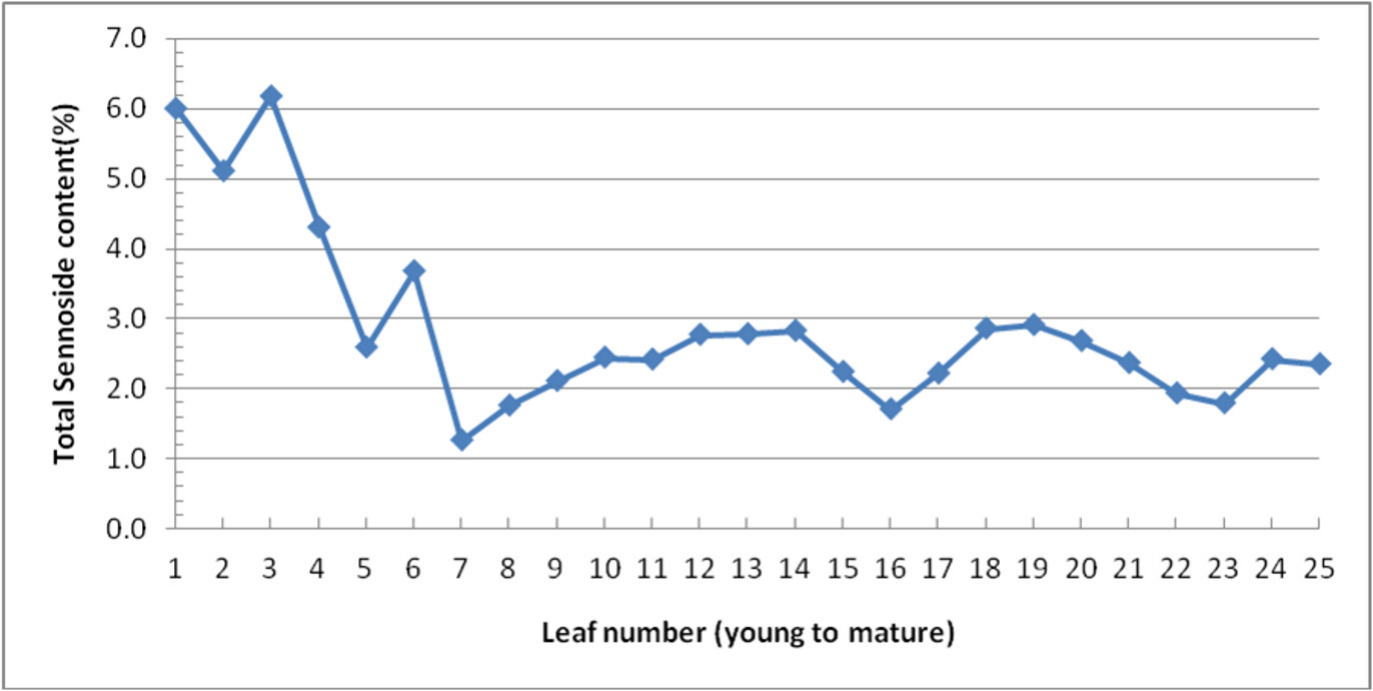
Variation in the total sennoside content (%) with ontogeny of the leaves.

High Performance Liquid Chromatography (HPLC) was used to estimate the total sennoside content in senna leaves using methanolic extract. UV-visible absorption spectrum of both standard sennoside and the leaf extract was recorded at 270 nm. A five level calibration curve was established over the range 0.168–1.68 mg/ml for sennoside-A (SA) and 0.38–7.56 mg/ml for sennoside-B (SB). The calibration curve obtained was Y = (9.196e–07) X+ (-0.0045) for SA and Y = (6.783e -07) X + (0.0747) for SB. Coefficient of correlation (R^2^) was 0.9997 for SA and 0.9969 for SB indicating good linearity of the curve. The chromatograms of the standard sennoside and senna leaf methanolic extract recorded peaks corresponding to sennoside are presented in S8 Fig. The first leaf (young; just opened) recorded 6.0% (w/w) sennoside content, second leaf (two day old) recorded 5.0% (w/w) sennoside content, and third leaf (three days old) recorded 6.2% (w/w) sennoside content which was the highest, whereas the seventh leaf from the top recorded 1.3% which was lowest (Fig 10). The sennoside content of other leaves (8^th^ to 25^th^ leaves) was in the range of 1.5 to 3.0% indicating the variation in the sennoside content with the leaf age.

## Discussion

Sennosides are the natural products of pharmaceutical importance. They have been used as natural, safe time-tested laxatives in traditional as well as modern systems of medicine. Variation in the total sennoside content (%) with ontogeny of the leaves was observed in the present study. Sennosides content was highest in youngest leaves. Decrease in sennosides content from youngest leaf to the leaf at the seventh node suggests that young leaf is an important tissue for the study of sennosides biosynthesis and transport. Earlier workers [5] also noticed higher concentration of sennosides in the youngest leaf and concentration decreased from youngest leaf to mature leaf. Although not quantified according to nodal position, other workers [64,65] have reported significantly higher sennosides content in the young leaves compared to mature leaves. The leaf sennoside content as 2.45% was reported [10] in senna. Higher Artemisinin content in the young leaves compared to mature leaves of *Artemisia annua* was reported [41]. Higher concentration of the terpenoid indole alkaloid, camptothecin (CPT) in *Camptotheca acuminata* was reported [66]. Higher sennosides content in the young leaves suggests, as in other higher plants containing secondary metabolites, that the sennoside precursors could be involved in some protective functions [67–69].

With availability of next-generation sequencing, medicinal plant transcriptome sequences are appearing in increasing numbers. The transcriptome study of the pathways leading to production of natural products such as sennosides in senna will help discover additional natural products for developing new drugs and manipulate pathways in plants and reconstitute plant pathways in microbial hosts. Next generation sequencing, as a high throughput as well as cost effective approach of sequence determination, has dramatically improved the efficiency and speed of gene discovery. Senna is an important plant used for medicinal purposes. Despite its medicinal importance, the transcriptome and genome information of senna are not available in the National Center for Biotechnology Information (NCBI) database. This work is the first application of the high-throughput RNA-Seq method to functionally annotate and quantify the expression levels of the transcriptome of the non-model plant system *C*. *angustifolia*, with an aim to understand the genes underlying the biosynthetic pathway of sennoside anthraquinones. We sequenced the transcriptome of young and mature leaves which differed for sennoside content. High sennoside content was recorded in young leaves compared to mature leaves. The two (young and mature) sequenced libraries were assembled and annotated separately using various bioinformatics tools. Although there might be genes of low abundance or conditionally expressed genes absent in this dataset, this study presents the most abundant genetic resource concerning the important medicinal plant *Cassia angustifolia*. We obtained approximately 6.34 Gb of raw sequence data, which was processed and *de novo* assembled into contigs and further to transcripts. The quality of a *de novo* assembly is dependent on many factors, such as the type of assembler used for sequence assembly followed by the parameters like N_50_ value and coverage. Trinity assembler was used in the present study. Trinity combines three independent software modules: Inchworm, Chrysalis, and Butterfly, applied sequentially to process large volumes of RNA-seq reads. Trinity was used to assemble the RNA-Seq data of neem [70], raddish [71] and medicago [72]. In general, the assembly of plant genome is challenge owing to larger genome size, complex gene content, higher rate of repeats and hetrozygosity [73]. The 1C content of *Cassia angustifolia* was reported to be 1.80 pg [74] which is higher compared to its close relatives *C*. *ariculata* (0.73 pg) and *C*. *tora* (0.68 pg) [75]. *De novo* transcriptome assembly using short read sequences in the absence of a reference genome sequence is difficult due to inherent error rate in the short reads, which limits specificity in assembly and the complexity of the transcriptome with respect to alternative splice forms, allelic variants, close paralogs, close homologs, and limitations in definitive quality assessment methods for the assembly [76]. Specific care was taken to remove adaptors and low quality sequences which could interfere in assembly process resulting in imperfect assembly or truncated contigs. The N_50_ value of the assembled data was high and comparable to other plant transcriptome assemblies, indicating a high quality assembly. Higher the N_50_ value better is the assembly. The N_50_ in our assembly was higher than most other published plant transcriptome assemblies [77]. The assembly results indicated that average transcript length of 1119 bp and 1467 bp in young and mature leaf transcriptome sequences, respectively, was longer than assembly of previously studied medicinal plants such as *Euphorbia fischeriana* [78], *Picrorhiza kurrooa* [47], *Chlorophytum borivilianum* [49] and *Costus pictus* [79]. We obtained 100% high quality bases for both young and mature leaves which indicate the high quality sequencing run. The results infer that the sequencing data of the transcriptome of *C*. *angustifolia* was most effectively assembled, which was further validated by the high proportion of CDS matched with known proteins and PCR amplification of SSRs. The average GC content of *C*. *angustifolia* transcripts was 42.31%, which was approximately equal to that of *Arabidopsis* (42.5%) and much lower than rice (55%) (monocot) in agreement with those reported earlier for monocots and dicots [80].

Functional annotation and classification provide information on cell metabolic pathways and biological behaviors of genes in the organism. The CDS annotated against the NCBI ‘green plant database (txid 33090)’ was utilized to assign functional GO annotation in terms of biological process, molecular function, and cellular groups. A large number of diverse GO assignments to CDS from this study highlight the diversity of genes likely represented in the leaf transcriptome and their involvement in many metabolic pathways while reflecting the global landscape of the transcriptome. The CDS without hits may belongs to untranslated regions, noncoding RNA, short sequence which does not contains protein domain or assembly mistakes. Maximum GO terms were assigned for molecular function category in young and mature leaf indicating need for large number of CDS for cell molecular functional activity. Maximum GO terms were assigned for molecular function category in medicinal plants such as *Hypericum perforatum* [48] and *Costus pictus* [79]. In the biological process category, most of the CDS were associated with “cellular processes” followed by “metabolic process” in young and mature leaves which may allow for the identification of novel genes involved in the secondary metabolite pathways. Reports on *Cassia obtusifolia* transcriptome, a member of the same family, also represents with the “cellular processes” followed by “metabolic process” of the transcripts in biological process category [81]. In the celluar component category, highest number of CDS was associated with “cell” and “cell part” followed by organelle in young and mature leaf samples which indicates the need of large number of transcripts for cell structure and maintaince. Under the molecular function category, the largest number of CDS was grouped in the “catalytic activity” followed by “binding activity” and “transporter activity” indicates the dominance of gene regulation, signal transduction, and enzymatically active processes in the cell. Maximum GO categories for catalytic activity and binding activity were also reported in *Glycyrrhiza uralensis* transcriptome [46]. In *Picrorhiza kurrooa*, transcripts of genes involved in DNA binding, catalytic and transferase activity were highly represented [47].

We used InterProScan to see shared conserved structural domains in the predicted proteins. Pentatricopeptide repeat (PPR) domain containing proteins represented the most in the senna leaf transcriptome indicating strong signal transduction mechanisms. Pentatricopeptide repeat containing proteins are a family of proteins commonly found in the plant kingdom [82]. They are involved in RNA editing and signal transduction with mitochondria and other organelles in plants [83]. PPR domain containing proteins are also represented most in the leaf transcriptome of *Physalis peruviana* [84]. Tetratricopeptide repeat (TPR) 14 domains represented next to PPR domains also have significance in signal transduction [85]. Leucine rich repeats (LRR) and WD40 are the other frequently occurring domains in the transcripts. Leucine-rich repeats are frequently involved in the formation of protein–protein interactions [86]. WD40-repeat proteins are a large family found in all eukaryotes and are implicated in a variety of functions ranging from signal transduction and transcription regulation to cell cycle control, autophagy, and apoptosis [87] indicating dominant protein domains in the leaf transcriptome that are of evolutionary significance.

Transcription factors regulate gene expression in response to various external and internal cues by activating or suppressing downstream genes. While performing the annotation analyses of the transcriptome data of senna, we have identified several transcription factors belonging to different families. Many transcripts were found to have the putative transcription factor encoding regions and have not been assigned to any particular transcription factor family. The most abundant families annotated include C3H, bHLH, MADS and MYB families in the leaf transcriptome. These transcription factors families known to regulate secondary metabolism play important role in control of anthraquinone biosynthesis [88–92]. C3H transcription factors belong to zinc finger motifs transcription factors family which play critical roles in interactions with other molecules [93–94]. C3H proteins are a large family containing zinc finger C3H-type motifs, and considerable evidence indicates that they are RNA-binding proteins that function in RNA processing [95–97]. MYB families are involved in various physiological programs like disease resistance, biotic and abiotic stress responses, developmental processes, growth, and senescence [98]. The presence of these proteins displayed in the transcriptome data and early analyses of the annotation results would guide further gene selection and functional experiments for their detailed characterization.

Senna is widely known for its pharmaceutical important sennoside anthraquinones and hence, gaining insights into the biosynthesis of sennosides and the transcriptional regulation of anthraquinones in general could accelerate the engineering of this pathway for production of high sennosides content in the near future. Using KEGG mapping of the best hit CDS, we have identified large number of CDS involved in metabolism, genetic information processing, environmental information processing, cellular processes and organizational systems. All these CDS are important resources for genetic manipulations of senna in the future. The metabolic pathways leading to sennoside are not known and little information is available on biosynthesis of anthraquinones in plants [15,20,50]. The anthraquinones are biosynthesized through combination of shikimate pathway (also isochorismate pathway) [19,20] and also through polyketide pathway [99]. The backbone of anthraquinones is synthesized via the isochorismate and MVA/MEP pathway [15]. The rings of A and B of anthraquinones are derived from 1,4-dihydroxy-2- naphthoic acid via isochorismic acid and α-ketoglutaric acid, whereas ring C of anthraquinones is derived from isopentenyl diphosphate (IPP)/3,3-dimethylallyl diphosphate (DMAPP) via the MVA/MEP pathway. Most of the genes encoding enzymes involved in the biosynthesis of the anthraquinone were present in the leaf transcriptome of senna in our study. There were more than one CDS assigned to the same enzyme. Such CDS may represent different fragments of a single transcript, different members of a gene family, or both. These results also demonstrated the powerful ability of high-throughput sequencing to identify genes in metabolic pathways. Transcriptome sequencing has been used to elucidate the biosynthetic pathways of Anthraquinones in *Cassia obtusifolia* [81], a close relative of senna and *Ophiorrhiza pumila* [50]. There are three rate limiting steps, which are catalyzed by Isopentenyldiphosphate isomerase (IPPS), 1-deoxy-Dxylulose-5-phosphate synthase (DXS), and isochorismate synthase (ICS), respectively, in the early stage of anthraquinones formation [100–102]. We have found that genes encoding *Acetyl-CoA C-acetyltransferase* (ACAT; EC 2.3.1.9), *Hydroxymethylglutaryl-CoA synthase* (HMGS; EC 2.3.3.10), *Hydroxymethylglutaryl-CoA reductase* (HMGR; EC 1.1.1.34), *Diphosphomevalonate decarboxylase* (MVD; EC 4.1.1.33), *1-deoxy-D-xylulose-5-phosphate synthase* (DXPS; EC 2.2.1.7), *2-C-methyl-D-erythritol 4-phosphate cytidylyltransferase* (ISPD; EC 2.7.7.60), *Isopentenyl-diphosphate delta-isomerase* (IPP; EC 5.3.3.2), *3-deoxy-7-phosphoheptulonate synthase* (DAHPS; EC:2.5.1.54), *3-dehydroquinate synthase* (DHQS; EC:4.2.3.4), *Menaquinone-specific isochorismate synthase* (menF; EC:5.4.4.2) and *Naphthoate synthase* (menB; EC:4.1.3.36) involved in the sennoside biosynthesis were deferentially expressed in young leaf compared to mature leaf, which suggest that these steps may be rate limiting in the formation of dimethylallyl diphosphate leading to anthraquinone formation. These genes form likely candidates for genetic manipulation of sennoside biosynthesis in senna. The enzymes 1-deoxy-D-xylulose-5-phosphate synthase (DXPS; EC 2.2.1.7), 4-diphosphocytidyl-2-C-methyl-D-erythritol kinase (CDPMEK; EC 2.7.1.148), (E)-4-hydroxy-3-methylbut-2-enyl-diphosphate synthase (HDS; EC 1.17.7.1) and 4-hydroxy-3-methylbut-2-enyl diphosphate reductase (HDR; EC 1.17.1.2) were highly differentially expressed in the hairy roots cultures compared to suspension cultures of *O*. *pumila* [50]. Functional characterization of the candidate genes will not only help elucidate the biochemical mechanism for life saving compounds biosynthesis, but also provide a molecular and biochemical target for improving the content of these compounds in future. Further enzyme assays of these enzymes are required to identify the function of the candidate genes. In plants, heme-containing CYPs are a super family of monoxygenases that catalyze the addition of oxygen atom to the metabolites and many of them are involved in plant secondary metabolism. CYPs are known to be involved in a wide range of biosynthetic pathways in medicinal plants, including those leading to the synthesis of glycyrrhizin [103], camptothecin [66], ginkgolide and flavonoid [104], tanshinone and salvianolic acid [105], lycopodium alkaloids [106], and picorosides [47]. The CYPs medicate many modifications of the backbone of anthraquinones in the later stage of anthraquinone biosynthesis [107,49]. In present study, 177 and 121 CDS in young and mature leaf, respectively, were identified as putative CYPs using BLAST search and 42 of them were found differentially expressing in young leaf as compared to mature leaf. Among them, CYP85A and CYP90B1 involved in brassinolide pathway [108–109] and CYP72A1, involved in monoterpenoid biosynthesis [110], are suggested to be possible candidates associated with sennoside biosynthesis. In the seed transcriptome *C*. *obtusifolia*, 30 CYPs, 12 SAM dependent methyltransferases, and 14 UDP-glucosyltransferase unigenes were identified [81]. These genes are therefore promising candidates for catalyzing the modifications of the anthraquinone which may also be involved in the biosynthesis of active metabolites.

Apart from gene discovery, transcriptomes also serve as invaluable reservoirs for discovery of SSRs, whose discovery earlier depend on the availability of DNA sequence [111]. SSRs are tandemly arranged repeats, ubiquitous, and found in both protein coding and non-coding regions affecting gene expression. They are favoured for a variety of applications in plant breeding because of their multi-allelic nature, reproducibility, codominant inheritance, high abundance, and extensive genome coverage [53]. These markers are used in high-throughput genotyping and thus, are used in development of high-density genetic maps, gene mapping, and marker-assisted selection. There is no information available on the nature and frequency of SSRs in senna. SSR markers designed from coding regions (transcriptomes) are more conserved compared to genomic SSRs and therefore show more transferability between species [54,112]. A total of 66,610 SSRs were identified from 43413 transcripts of *C*. *angustifolia*. There was one SSR per 1.08 Kb of transcriptome sequence-a frequency higher than that reported earlier in plants [45,113]. Most abundant repeat motifs found in the present study were hexanucleotides repeats which was in agreement with previous studies [114]. Trinucleotide repeat constitute the next most prevalent motifs after hexanucleotides motifs. This is similar to the earlier observations on the relative abundance of trinucleotide motifs in the EST sequences of cereals [114,115] and other plant genome [116]. Higher frequency of the trinucleotide repeat motifs than the other classes could be attributed to the selection against frame shift mutations that limit expansion of non triplet microsatellites [117]. The difference in the frequencies of SSRs could be attributed to the ‘‘search criteria” used, type of SSR motif, size of sequence data analysis, and the mining tool used [112,113,118]. In the present study, 35 genic SSRs were designed and the PCR amplification of a set of 35 SSR markers revealed 62% successful amplification. We for the first time developed genic SSR makers for *C*. *angustifolia* which will have applications in functional diversity studies, association mapping studies, QTL mapping studies for sennoside content and other economic traits in senna.

In summary, senna is a suitable medicinal herbal model for investigating sennoside biosynthesis, but without genome-scale information. Here, the transcriptome annotation presents the most abundant genetic resource for *Cassia angustifolia* to date. It will serve as the foundation for other functional genomic research efforts and genetic engineering to improve the production of active principal compounds.

## Supporting Information

S1 Fig Per base sequence quality score (Phred) of leaf transcriptome of *Cassia angustifolia*. (DOCX)

S2 Fig Transcript size (bp) distribution in the assembled leaf transcriptome of *Cassia angustifolia*. (DOC)

S3 Fig ATGC composition of assembled transcripts of *Cassia angustifolia* leaf transcriptome A) young and B) mature leaf transcripts. (DOC)

S4 Fig Size distribution of CDS in the leaf transcriptome of Cassia angustifolia. (DOC)

S5 Fig BLASTX top hit species distribution of transcript contigs in the leaf transcriptome of *C*. *angustofolia*. A) young and B) mature leaf. (DOC)

S6 Fig GO Classification. GO terms were derived based on the similarity search with in young and mature leaf CDS in the transcriptome of *Cassia angustofolia*.
(DOC)

S7 Fig Amplification of Genic-SSR marker Xcadas11 developed in the present study in 48 germplasm accessions of Senna. (PPTX)

S8 Fig HPLC chromatogram of leaf extracts of *Cassia angustifolia* (I) and reference standards sennoside-A and sennoside-B (II). (DOC)

S1 Table KEGG pathways in young leaf of *Cassia angustifolia*. (XLS)

S2 Table KEGG pathways in mature leaf of *Cassia angustifolia*. (XLS)

S3 Table Differential expression of genes involved leaf transcriptome of *Cassia angustifolia*. (XLS)

S4 Table Gene ontology (GO) enrichment analysis of 2,343 CDS up-regulated in young leaf compared to mature leaf transcriptome of *Cassia angustifolia*. (XLSX)

S5 Table Differentially expressed annotated Cytochrome P450s (CYPs) in leaf transcriptome of *C*. *angustifolia*. (XLS)

S6 Table Distribution of SSRs in *Cassia angustifolia* leaf Transcriptome. (DOC)

S7 Table Details of 22 genic-SSRs markers developed in this study using leaf transcriptome sequence of *Cassia angustifolia*. (DOC)
