# Supplementary material for: Next Generation Sequencing and Transcriptome Analysis Predicts Biosynthetic Pathway of Sennosides from Senna (Cassia angustifolia Vahl.), a Non-Model Plant with Potent Laxative Properties
Source: PLoS One. 2015 Jun 22;10(6):e0129422. doi: 10.1371/journal.pone.0129422 (PMC4476680; doi:10.1371/journal.pone.0129422)
Supplement: S1 Fig — (DOCX) [file pone.0129422.s001.docx]

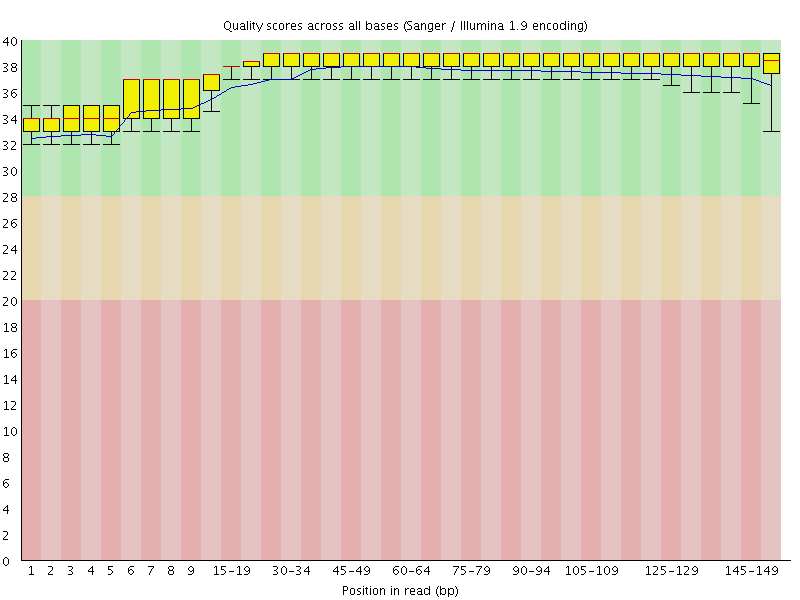
**Figure S6a. Per base sequence quality score (Phred) of young leaf transcriptome of *Cassia angustifolia***

**
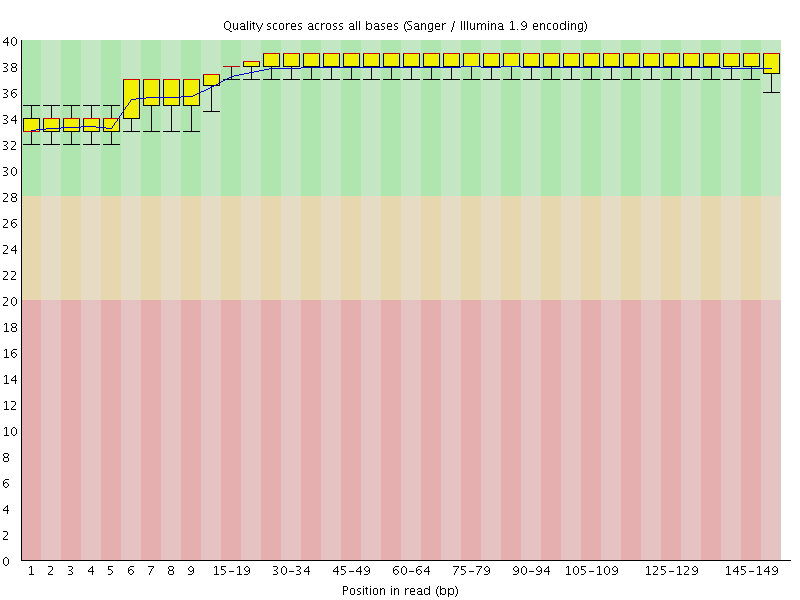
Figure S6b. Per base sequence quality score (Phred) of mature leaf transcriptome of *Cassia angustifolia***
