# Supplementary material for: Next Generation Sequencing and Transcriptome Analysis Predicts Biosynthetic Pathway of Sennosides from Senna (Cassia angustifolia Vahl.), a Non-Model Plant with Potent Laxative Properties
Source: PLoS One. 2015 Jun 22;10(6):e0129422. doi: 10.1371/journal.pone.0129422 (PMC4476680; doi:10.1371/journal.pone.0129422)
Supplement: S3 Fig — (DOC) [file pone.0129422.s003.doc]

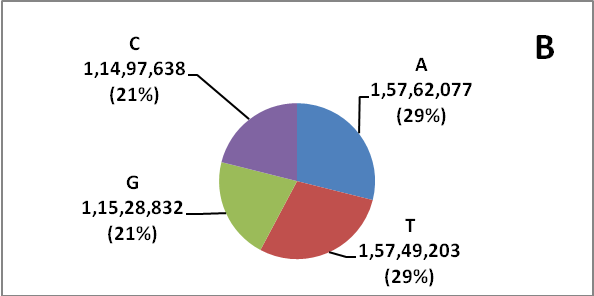

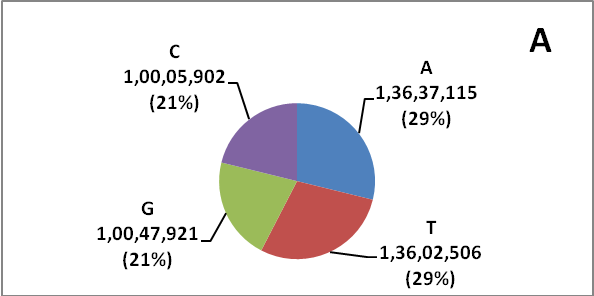


**Figure S3: ATGC composition of assembled transcripts of *Cassia angustifolia* leaf transcriptome A) young and B) mature leaf transcripts**
