# Supplementary material for: Next Generation Sequencing and Transcriptome Analysis Predicts Biosynthetic Pathway of Sennosides from Senna (Cassia angustifolia Vahl.), a Non-Model Plant with Potent Laxative Properties
Source: PLoS One. 2015 Jun 22;10(6):e0129422. doi: 10.1371/journal.pone.0129422 (PMC4476680; doi:10.1371/journal.pone.0129422)
Supplement: S5 Fig — (DOC) [file pone.0129422.s005.doc]

**
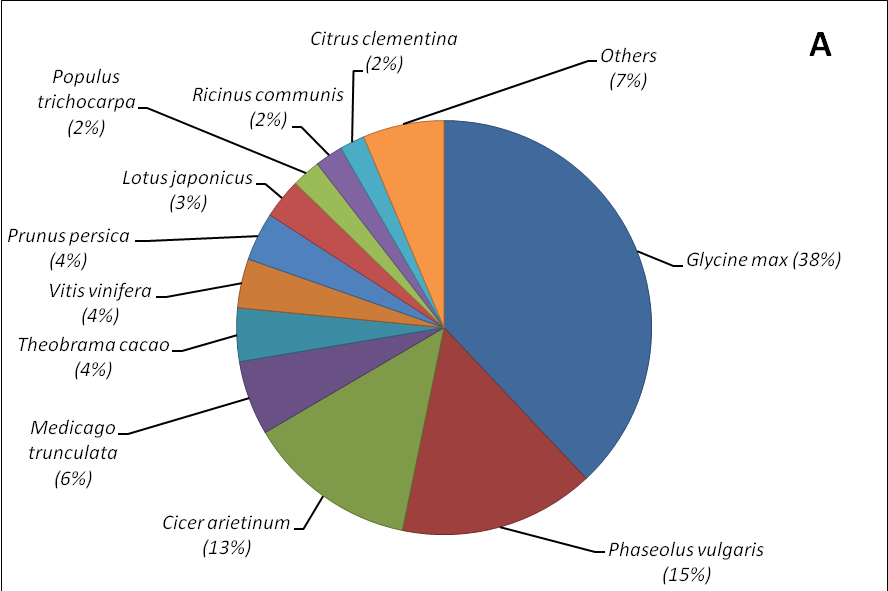
**

**
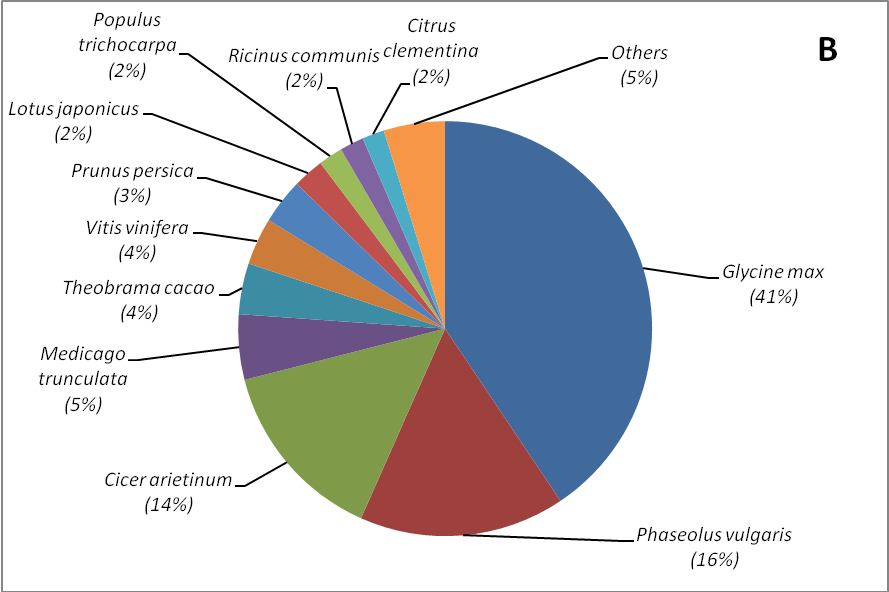
**

**Figure S5. BLASTX top hit species distribution of transcript contigs in the leaf transcriptome of *C*. *angustofolia.* A) young and B) mature leaf**
