# Supplementary material for: Next Generation Sequencing and Transcriptome Analysis Predicts Biosynthetic Pathway of Sennosides from Senna (Cassia angustifolia Vahl.), a Non-Model Plant with Potent Laxative Properties
Source: PLoS One. 2015 Jun 22;10(6):e0129422. doi: 10.1371/journal.pone.0129422 (PMC4476680; doi:10.1371/journal.pone.0129422)
Supplement: S6 Fig — GO terms were derived based on the similarity search with in young and mature leaf CDS in the transcriptome of Cassia angustofolia. (DOC) [file pone.0129422.s006.doc]

**
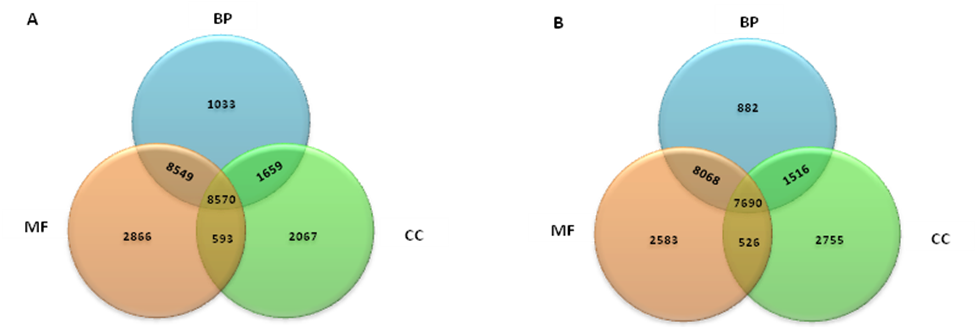
**

**Figure S6. GO obtained in A) young and B) mature leaf transcriptome of *Cassia angustifolia.*** GO terms classified into biological process (BP), molecular function (MF) and cellular process (CC).
