# Supplementary material for: Next Generation Sequencing and Transcriptome Analysis Predicts Biosynthetic Pathway of Sennosides from Senna (Cassia angustifolia Vahl.), a Non-Model Plant with Potent Laxative Properties
Source: PLoS One. 2015 Jun 22;10(6):e0129422. doi: 10.1371/journal.pone.0129422 (PMC4476680; doi:10.1371/journal.pone.0129422)
Supplement: S7 Fig — (PPTX) [file pone.0129422.s007.pptx]

## Slide 1
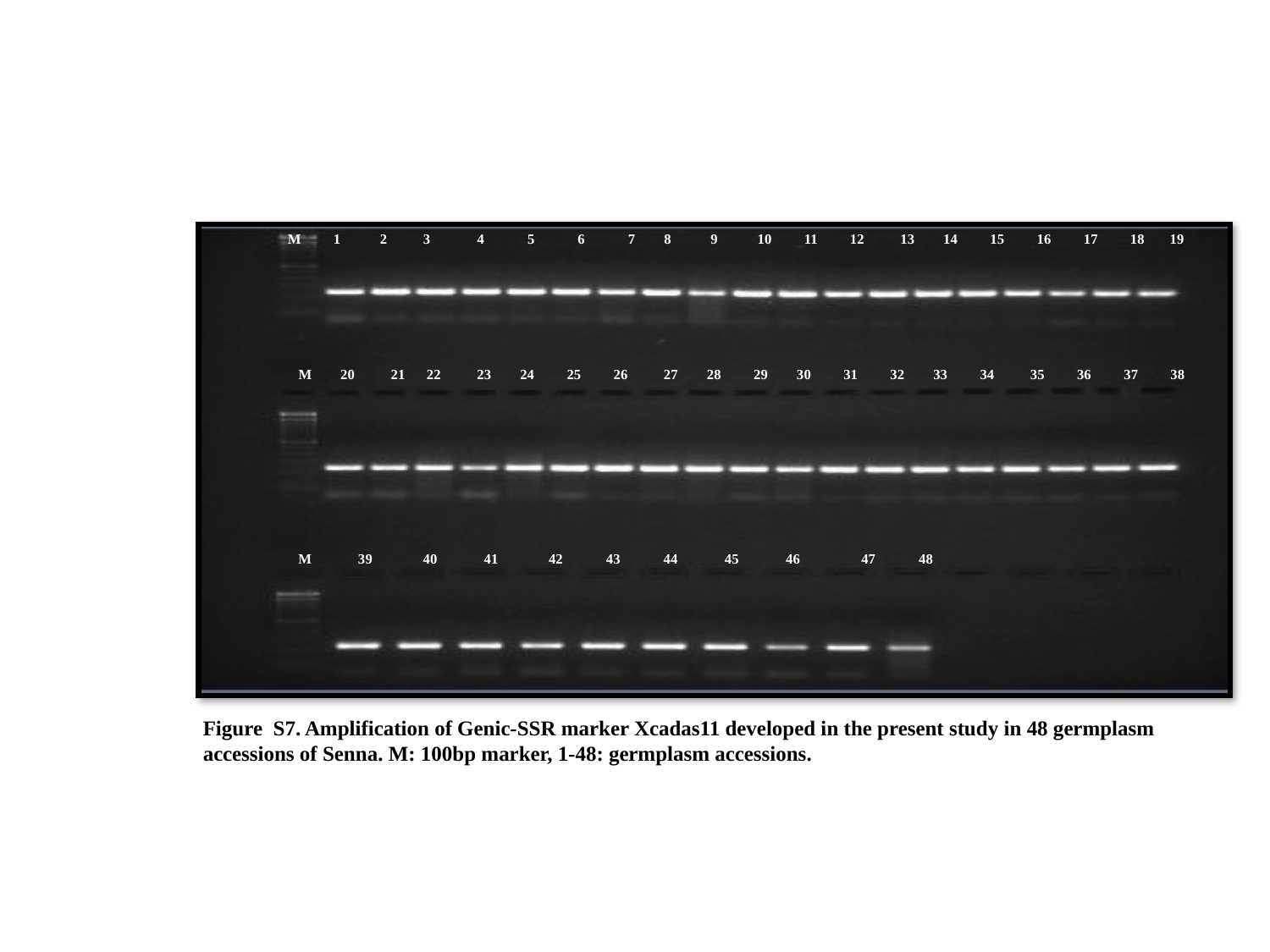

M 1 2 3 4 5 6 7 8 9 10 11 12 13 14 15 16 17 18 19
 M 20 21 22 23 24 25 26 27 28 29 30 31 32 33 34 35 36 37 38
M 39 40 41 42 43 44 45 46 47 48
Figure S7. Amplification of Genic-SSR marker Xcadas11 developed in the present study in 48 germplasm accessions of Senna. M: 100bp marker, 1-48: germplasm accessions.
