# Supplementary material for: Next Generation Sequencing and Transcriptome Analysis Predicts Biosynthetic Pathway of Sennosides from Senna (Cassia angustifolia Vahl.), a Non-Model Plant with Potent Laxative Properties
Source: PLoS One. 2015 Jun 22;10(6):e0129422. doi: 10.1371/journal.pone.0129422 (PMC4476680; doi:10.1371/journal.pone.0129422)
Supplement: S8 Fig — (DOC) [file pone.0129422.s008.doc]

I

**Time [Minutes]**

II

**Time [Minutes]**

**Figure S8: HPLC chromatogram of leaf extracts of *Cassia angustifolia* (I) and reference standards sennoside-A and sennoside-B (II)**
