# Supplementary material for: Next Generation Sequencing and Transcriptome Analysis Predicts Biosynthetic Pathway of Sennosides from Senna (Cassia angustifolia Vahl.), a Non-Model Plant with Potent Laxative Properties
Source: PLoS One. 2015 Jun 22;10(6):e0129422. doi: 10.1371/journal.pone.0129422 (PMC4476680; doi:10.1371/journal.pone.0129422)
Supplement: S6 Table — (DOC) [file pone.0129422.s014.doc]

**Table S6**. **Distribution of SSRs in *Cassia angustifolia* leaf transcriptome**

| **SSR Type** | **Motif Type** | **Motif length (Repeat unit)** | | | | | | | | | | | | | |
| --- | --- | --- | --- | --- | --- | --- | --- | --- | --- | --- | --- | --- | --- | --- | --- |
|  |  | **2** | **3** | **4** | **5** | **6** | **7** | **8** | **9** | **10** | **11** | **12** | **Total** | **Motif (%)** | **Total (%)** |
| **Dimers** | **AC/GT** | - | - | - | - | 135 | 126 | 30 | 94 | 26 | 7 | 3 | 421 | 10.98 |  |
|  | **AG/CT** | - | - | - | - | 670 | 406 | 520 | 336 | 310 | 133 | 9 | 2384 | 62.20 |  |
|  | **AT/AT** | - | - | - | - | 325 | 180 | 139 | 133 | 177 | 57 | 3 | 1014 | 26.45 |  |
|  | **CG/CG** | - | - | - | - | 12 | 2 | - | - | - | - | - | 14 | 0.37 |  |
|  | **Total** | **-** | **-** | **-** | **-** | **1142** | **714** | **689** | **563** | **513** | **197** | **15** | **3833** |  | **5.75** |
| **Trimers** | **AAC/GTT** | - | - | 286 | 97 | 31 | 7 | - | - | - | - | - | 421 | 5.30 |  |
|  | **AAG/CTT** | - | - | 1414 | 466 | 355 | 180 | 20 | - | - | - | - | 2435 | 30.66 |  |
|  | **AAT/ATT** | - | - | 270 | 82 | 53 | 36 | 5 | - | - | - | - | 446 | 5.62 |  |
|  | **ACC/GGT** | - | - | 413 | 102 | 69 | 12 | 2 | - | - | - | - | 598 | 7.53 |  |
|  | **ACG/CGT** | - | - | 241 | 89 | 9 | 6 | 1 | - | - | - | - | 346 | 4.36 |  |
|  | **ACT/AGT** | - | - | 100 | 14 | 14 | - | - | - | - | - | - | 128 | 1.61 |  |
|  | **AGC/CTG** | - | - | 617 | 141 | 53 | 26 | 5 | - | - | - | - | 842 | 10.60 |  |
|  | **AGG/CCT** | - | - | 602 | 250 | 73 | 73 | - | - | - | - | - | 998 | 12.56 |  |
|  | **ATC/ATG** | - | - | 791 | 366 | 110 | 55 | 6 | - | - | - | - | 1328 | 16.72 |  |
|  | **CCG/CGG** | - | - | 281 | 82 | 34 | 3 | 1 | - | - | - | - | 401 | 5.05 |  |
|  | **Total** | **-** | **-** | **5015** | **1689** | **801** | **398** | **40** | **-** | **-** | **-** | **-** | **7943** | **-** | **11.92** |
| **Tetramers** | **0% AT** | - | 8 | - | - | - | - | - | - | - | - | - | 8 | 0.21 |  |
|  | **25%AT** | - | 218 | 30 | 3 | 7 | - | - | - | - | - | - | 258 | 6.79 |  |
|  | **50%AT** | - | 963 | 94 | 33 | 5 | - | - | - | - | - | - | 1095 | 28.82 |  |
|  | **75%AT** | - | 1514 | 219 | 86 | 29 | - | - | - | - | - | - | 1848 | 48.63 |  |
|  | **100%AT** | - | 494 | 81 | 16 | - | - | - | - | - | - | - | 591 | 15.55 |  |
|  | **TOTAL** | **-** | **3197** | **424** | **138** | **41** | **-** | **-** | **-** | **-** | **-** | **-** | **3800** |  | **5.70** |
| **Pentamers** | **0% AT** | - | 17 | - | - | - | - | - | - | - | - | - | 17 | 1.84 |  |
|  | **20%AT** | - | 20 | - | - | - | - | - | - | - | - | - | 20 | 2.16 |  |
|  | **40%AT** | - | 133 | 17 | 8 | - | - | - | - | - | - | - | 158 | 17.06 |  |
|  | **60%AT** | - | 208 | 42 | - | - | - | - | - | - | - | - | 250 | 27.00 |  |
|  | **80%AT** | - | 306 | 47 | 2 | - | - | - | - | - | - | - | 355 | 38.34 |  |
|  | **100%AT** | - | 108 | 18 | - | - | - | - | - | - | - | - | 126 | 13.61 |  |
|  | **Total** | - | 792 | 124 | 10 | - | - | - | - | - | - | - | **926** |  | **1.39** |
| **Hexamers** | **0% AT** | 91 | 12 | - | - | - | - | - | - | - | - | - | 103 | 0.21 |  |
|  | **16.6%AT** | 1223 | 82 | 25 | - | - | - | - | - | - | - | - | 1330 | 2.65 |  |
|  | **33.3%AT** | 5772 | 236 | 89 | - | - | - | - | - | - | - | - | 6097 | 12.17 |  |
|  | **50%AT** | 15992 | 645 | 94 | - | - | - | - | - | - | - | - | 16731 | 33.39 |  |
|  | **66.6%AT** | 15387 | 291 | 49 | - | - | - | - | - | - | - | - | 15727 | 31.39 |  |
|  | **83.3%AT** | 8362 | 176 | 17 | - | - | - | - | - | - | - | - | 8555 | 17.07 |  |
|  | **100%AT** | 1546 | 17 | 2 | - | - | - | - | - | - | - | - | 1565 | 3.12 |  |
|  | **Total** | 48373 | 1459 | 276 | - | - | - | - | - | - | - | - | **50108** |  | **75.23** |
|  | **Total** | **48373** | **5448** | **5839** | **1837** | **1984** | **1112** | **729** | **563** | **513** | **197** | **15** | **66610** |  |  |
