# Supplementary material for: Next Generation Sequencing and Transcriptome Analysis Predicts Biosynthetic Pathway of Sennosides from Senna (Cassia angustifolia Vahl.), a Non-Model Plant with Potent Laxative Properties
Source: PLoS One. 2015 Jun 22;10(6):e0129422. doi: 10.1371/journal.pone.0129422 (PMC4476680; doi:10.1371/journal.pone.0129422)
Supplement: S7 Table — (DOC) [file pone.0129422.s015.doc]

**Table S7. Details of 22 Genic- SSRs markers developed in this study using leaf transcriptome sequence of *Cassia angustifolia***

| **Marker** | **Transcript** | **Repeat motif** | **Forward primer (5’→3’)** | **Reverse primer (5’→3’)** | **T_m_ (^o^C)** | **Expected**  **amplicon**  **size (bp)** | **Putative function** |
| --- | --- | --- | --- | --- | --- | --- | --- |
| Xdacaem02 | transcript_contig_973 | (AGA)_8_ | GGAGTTGTCGGAGTGGTAGT | TGCTGGATATGTGTTTGCGG | 48.5 | 243 | Unknown [*Lotus japonicus*] |
| Xdacaem04 | transcript_contig_85 | (GTG)_8_ | CAGCCTCCTCATGAATGTGC | GAGGGAGAGAGACAATGGCA | 54 | 278 | PREDICTED: phosphoribulokinase, chloroplastic-like [*Glycine max*] |
| Xdacaem05 | transcript_contig_83 | (ACC)_8_ | GAGGGAGAGAGACAATGGCA | CAGCCTCCTCATGAATGTGC | 54 | 278 | PREDICTED: phosphoribulokinase, chloroplastic-like [*Glycine max*] |
| Xdacaem07 | transcript_contig_4674 | (TCC)_8_ | CGATGGTAGTGCTTTGCCTC | AACGACCTGCAATGAACGAC | 48.5 | 287 | Hypothetical protein PHAVU_004G038800g [*Phaseolus vulgaris*] |
| Xdacaem08 | transcript_contig_4673 | (TCC)_8_ | CGATGGTAGTGCTTTGCCTC | AACGACCTGCAATGAACGAC | 48.5 | 287 | Hypothetical protein PHAVU_004G038800g [*Phaseolus vulgaris*] |
| Xdacaem10 | transcript_contig_4461 | (AT)_11_ | TGCTTGGAGGGGAGATTGTT | GCACCCCTCTCACCTAATCA | 48.5 | 200 | Hypothetical protein PHAVU_011G077900g [*Phaseolus vulgaris*] |
| Xdacaem11 | transcript_contig_44 | (GTC)_8_ | CCTGGAAGATTCAGTCCCGT | GCTTTGAGGGGCACTTTTGA | 51 | 249 | Chlorophyll A/B binding protein, putative [*Ricinus communis*] |
| Xdacaem14 | transcript_contig_380 | (AT)_11_ | GGTCAATCTGGAAGTGGGGT | TGGGTTCCAGCATTCCTCTG | 46 | 243 | Aquaporin PIP11 [*Medicago truncatula*] |
| Xdacaem15 | transcript_contig_29121 | (ATC)_8_ | TCTTCGCATCATCAGCCTCT | TGCTGCTTCATCTTCTGTGC | 46 | 274 | hypothetical protein PRUPE_ppa012494mg [*Prunus persica*] |
| Xdacaem16 | transcript_contig_278 | (AG)_11_ | CCGCCCATGATTTTCCTGTT | TTGGAGTCATGGAAGCTGGA | 46 | 277 | PREDICTED: thiol protease aleurain-like isoform 1 [*Vitis vinifera*] |
| Xdacaem19 | transcript_contig_2208 | (TGA)_8_ | TCCCCTTTCATGAACCAGAGA | GGATTTGTGGGTTGTGTGCT | 54 | 288 | Hypothetical protein PHAVU_010G128400g [*Phaseolus vulgaris*] |
| Xdacaem20 | transcript_contig_1871 | (TC)_11_ | AGCCCCTAACCCTTTTCTGC | TCGTCATTCTTGGGGTTCGA | 46 | 289 | Unknown [*Lotus japonicus*] |
| Xdacaem21 | transcript_contig_18580 | (TG)_11_ | CTTCTCTGTCACGAGCGTTG | TGGAGGAACGTCTGGAAACT | 46 | 271 | PREDICTED: uncharacterized protein LOC100267059 [*Vitis vinifera*] |
| Xdacaem22 | transcript_contig_18205 | (CT)_11_ | TGGCCCTTCCCCTTAAAGAA | GAGTGGGTGTGGTAGTGACA | 46 | 248 | Unknown |
| Xdacaem23 | transcript_contig_16345 | (GGA)_8_ | AACGACCTGCAATGAACGAC | CGATGGTAGTGCTTTGCCTC | 46 | 287 | Hypothetical protein PHAVU_004G038800g [*Phaseolus vulgaris*] |
| Xdacaem26 | transcript_contig_12749 | (CT)_11_ | TCTGGGTTAGGGTCTTGAGC | ACGAAGCTCCCACACACTAT | 46 | 240 | Hypothetical protein CICLE_v10029527mg [*Citrus clementina*] |
| Xdacaem27 | transcript_contig_126 | (TC)_11_ | TATCTCCCTCGCTTCCCTCA | TAGAAGCAGCCTTGACGACA | 54 | 247 | Type III chlorophyll a/b-binding protein [*Lycoris aurea*] |
| Xdacaem28 | transcript_contig_12376 | (GGA)_8_ | AACGACCTGCAATGAACGAC | CGATGGTAGTGCTTTGCCTC | 51 | 287 | Hypothetical protein PHAVU_004G038800g [*Phaseolus vulgaris*] |
| Xdacaem29 | transcript_contig_1237 | (AG)_11_ | GCAGAACCGTACACCCTTTT | AGCTGGTCACAATCCTCCTC | 46 | 234 | PREDICTED: uncharacterized protein sll0005-like [*Glycine max*] |
| Xdacaem30 | transcript_contig_120 | (GTG)_8_ | GCTTCCTCTGCTTCTCTTGC | CAAACTTCGCCTCACAGCTT | 54 | 292 | PREDICTED: phosphoribulokinase, chloroplastic-like [*Glycine max*] |
| Xdacaem34 | transcript_contig_10744 | (AGC)_8_ | CTGCGAGATCACCAGAAACG | GAAACATGTCATCCCTGGGC | 54 | 252 | hypothetical protein PHAVU_010G132500g [*Phaseolus vulgaris*] |
| Xdacaem35 | transcript_contig_1057 | (AG)_11_ | GCAGAACCGTACACCCTTTT | AGCTGGTCACAATCCTCCTC | 45 | 234 | PREDICTED: uncharacterized protein sll0005-like [*Glycine max*] |
